# A moral Turing test: How belief and source shape detection of and agreement with LLM judgments

**DOI:** 10.1371/journal.pone.0353391

**Published:** 2026-08-05

**Authors:** Basile Garcia, Crystal Qian, Stefano Palminteri

**Affiliations:** 1 University of Geneva, Geneva, Switzerland; 2 Google DeepMind, New York, New York, United States of America; 3 Département d’études cognitives, École normale supérieure, PSL Research University, Paris, France; 4 Laboratoire de Neurosciences Cognitives Computationnelles, Institut National de la Santé et de la Recherche Médicale, Paris, France; University of Stavanger, NORWAY

## Abstract

As large language models (LLMs) are increasingly integrated into decision-making systems (e.g., autonomous vehicles and medical devices), understanding how humans perceive and evaluate AI-generated judgments is crucial. To investigate this, we conducted a series of experiments in which participants evaluated justifications for moral and non-moral choices, generated either by humans or LLMs. Participants attempted to identify the source of each justification (either human or LLM) and indicated their agreement with its content. We found that while detection accuracy was consistently above chance, it remained below 75%. In terms of agreement, there was no overall preference for human-generated responses, even though machine-generated justifications were favored in particularly challenging moral scenarios. Notably, we observed a systematic anti-AI bias: participants were less likely to agree with judgments they *believed* were AI-generated, regardless of the true source. Linguistic cues, such as response length, typos, first-person pronouns, and cost-benefit language markers (e.g., “lives,” “save”), influenced both detection and agreement. Participants tended to disagree with cost-benefit calculations, possibly due to an expectation that AI would favor such reasoning. These findings highlight the influence of motivated belief and ingroup/outgroup bias in shaping human evaluation of AI-generated content, particularly in morally sensitive contexts.

## Introduction

Large language models (LLMs) are becoming widely used in applications ranging from conversational agents to decision-making systems capable of consequential decisions, such as providing medical advice [1], legal advice [2] and mental well-being support [3]. In their daily lives, early adopters are already utilizing chatbots to inform their personal decision-making [4]. Moreover, a substantial proportion of individuals appear willing to delegate moral decisions to AIs [5]. As humans increasingly interact with LLMs, understanding our ability to detect and align with LLMs’ judgments becomes crucial, particularly given the risk of misuse, such as the dissemination of disinformation by LLM-powered bots [6,7].

Determining whether a decision is made by a human or a machine is crucial: it enhances safety by revealing our susceptibility to manipulation, possesses intrinsic epistemic value [8], and guides the development of LLMs towards human- preferred outputs [9]. Concerningly, recent studies indicate that humans often struggle to reliably distinguish AI-generated texts from human texts, across diverse contexts such as poetry [10–12] and media misinformation [13]. Additionally, strategies can be employed to “humanize” AI-generated content to increase the difficulty of detection. “Humanized” LLMs have sometimes been judged as more human-like than actual human-generated responses [14].

Before LLMs, research into applied fields such as autonomous vehicles highlighted the need for alignment in human and machine moral decision making [15]. Prior research has shown a human tendency to favor human-generated decisions over machine-generated ones, a phenomenon known as algorithm aversion [16]. However, this phenomenon is context-dependent [17]. For instance, humans tend to prefer human judgment over AI judgment in the context of medical decision-making [18], but prefer AI judgment in numerical tasks, such as song rank forecasts based on big data methods [19].

Agreement with LLM-generated text hinges on factors like task nature, perceived authorship [20], prior AI interactions, and even cultural context [21]. While ChatGPT’s responses in social scenarios have been rated as more balanced and empathetic than human advice [22–24], people still show a strong preference for human advice on moral issues [25]. AI authors are perceived as less competent, though humans still value their advice [26]. Moreover, in persuasive content creation (e.g., advertisements), AI efforts are often rated higher than human efforts. Revealing the source of content production lessens the quality gap between human- and AI-generated content, without affecting the assessed quality of AI-created content [27]. This suggests that human favoritism, rather than AI aversion, drives the perceived quality and perceived value [28].

Taken together, these findings suggest the human perception of machine judgment can go in two opposite directions. A preference for AI may occur when machines are seen as authoritative sources of knowledge. In contrast, an aversion for AI might emerge from societal or psychological prejudices against machines, often stemming from the belief that machines lack agency and the capacity for compassionate or morally sound decisions [29]. Ultimately, the importance of source identification depends heavily on the context of the interaction. Knowing whether content is AI- or human-generated becomes highly consequential when accountability is at stake, when the content is advisory rather than purely informational, and when users might calibrate their trust differently based on the author. While deferring to AI may be less problematic for pattern-recognition tasks with a clear ground truth, it becomes far more complex when the grounds for judgment are themselves contested. Moral preferences are precisely such a case: they capture ethically consequential scenarios [30,31] where the basis for judgment is inherently subjective, making them a critical testing ground for examining the detection and acceptance of machine judgments by humans.

Recent explorations into this moral testing ground have yielded mixed results. For instance, Scherrer et al. [32] find that LLMs generally align with human moral values, but in ambiguous cases, their responses can vary based on question phrasing, with closed-source models demonstrating more consistent preferences. On a day-to-day moral level, however, LLMs show judgment patterns that differ substantially from those of Reddit users [33]. This variation may arise from differences in pre-training data and fine-tuning processes [34,35]. These findings suggest that linguistic features play a significant role in assessing the moral quality of judgments from humans or AI.

However, while prior work has explored AI detection and alignment, the relationship between identification and agreement remains empirically under-investigated, especially in moral decision-making processes, because either the interaction was overlooked [36,37] or was not investigated in the moral domain [38]. Specifically, it remains unclear whether a participant’s belief about the source of content affects their agreement. Our research directly addresses this gap, exploring humans’ capacity to detect the source of moral judgments (either human or LLM), their agreement with these judgments, and critically, the relation between these two behavioral outcomes. Additionally, we explore the linguistic factors influencing identification and agreement [32,39].

### The present study

To investigate human perceptions of AI moral decision-making, we conducted a series of quantitative experiments involving 230 participants (N = 230). Full demographic details (age, gender, country) are reported in the Methods (see *Participants*). First, we collected a corpus of moral judgments by presenting 60 diverse ethical scenarios [40,41] to human participants and LLM models in the GPT-3.5 family [42,43]. These scenarios featured “personal” and “impersonal” moral dilemmas, many of which centered on a classic tradeoff between a utilitarian calculation and a strong emotional aversion (a deontological-like consideration). Personal moral scenarios were shown to create a high-conflict challenge for human participants, more so than impersonal ones [40,41]. We note that refusals in personal moral scenarios may reflect emotionally-driven aversion rather than principle-based (i.e., strictly deontological) reasoning; throughout the paper we reserve the term “deontological” for principle-based judgments and use “non-utilitarian” where the distinction matters. For instance, in the classic ‘trolley problem,’ participants decide whether to divert a runaway trolley to save five people at the cost of one (an impersonal dilemma). In the ‘footbridge’ variant, participants must decide whether to physically push a person off a bridge to stop the trolley (a personal dilemma; see Fig 2A for examples across scenario types).

**Fig 1:**
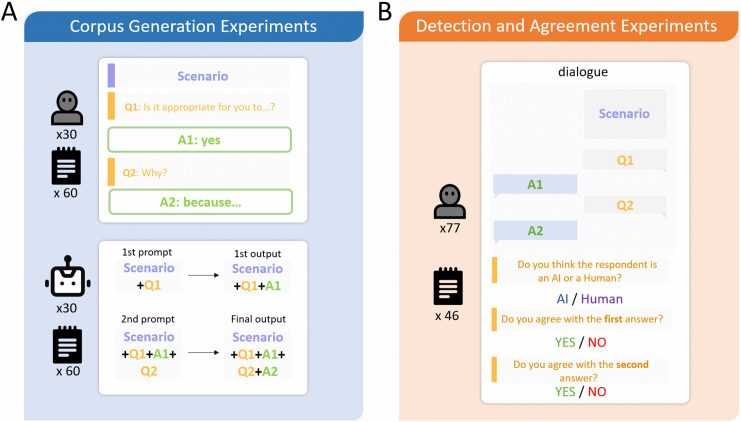
Schematic Experimental Flow and Interfaces for Corpus Generation and Detection Tasks. (A) Schematic interface and method used in the experiment we used to generate the dv2 and dv3 corpora. (B) Schematic interface used in the *detection and agreement task.* Robot icon by Konstantin Filatov, from the Gentlecons Interface Icons collection. Licensed under CC BY.

First, our “machine psychology” experiment revealed that, similar to humans, LLMs’ moral preferences exhibited strong contextual influences [40,44–46]. We then presented these judgments to new groups of participants, who were tasked with identifying the source (human or AI), expressing their agreement or disagreement with the judgment itself, as well as agreement with the accompanying justification. First, participants detected AI-generated justifications with moderate accuracy, especially in moral scenarios. Concerning their agreement, we found that, while participants favored human justifications in non-moral scenarios, they preferred LLM-generated justifications in challenging moral scenarios (like the footbridge problem). This may reflect a preference for deliberative reasoning in high-stakes situations. Critically however, the preference for LLM judgments concerning morally challenging scenarios was accompanied by a general false belief that the acceptable justifications came from humans. Finally, we analyzed the possible linguistic determinants of detection and agreement and the textual and semantic levels. In our data, low-level linguistic features (length, typos, first-person pronouns) predicted participants’ beliefs about the source but were not themselves associated with agreement; acceptability is thus better described as being mediated through source inference than as being a direct response to linguistic quality.

## Results

### Overview of the study

To systematically investigate human perceptions of AI moral decision-making, our study was conducted in three main phases. First, in the corpus generation phase, we collected judgments and textual justifications for a diverse set of moral and non-moral scenarios from both human participants and large language models (specifically, GPT-3.5 variants). Second, in the corpus evaluation phase, we presented these responses to new, independent groups of human evaluators who were tasked with identifying the authorship source (AI or human) and indicating their level of agreement with both the judgment and the justification. Finally, we performed extensive linguistic and semantic analyses, utilizing NLP techniques, predictive modeling, and SHAP interpretations, to isolate the specific textual features (such as formal reasoning cues and cost-benefit markers) that drove participants’ detection accuracy and moral alignment. Full methodological details are provided in the Methods section.

### LLMs replicate the moral framing effect

**Fig 2:**
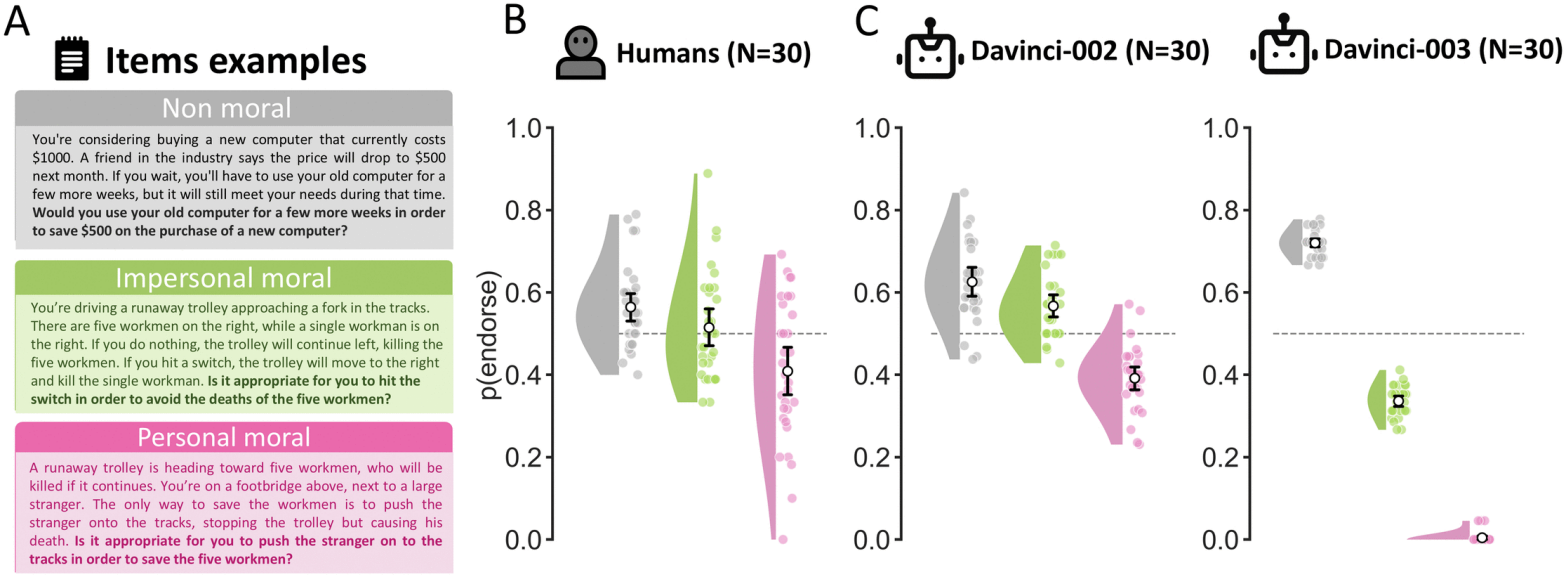
Moral Dilemma Scenarios and Endorsement Rates by Humans and LLMs. (A) Example of scenarios across three categories (taken from Greene et al. 2004). (B) Endorsement of the different moral actions as a function of category of scenario; ‘non moral’ refers to scenarios with no moral stakes; ‘impersonal moral’ refers to scenarios with moral scenario whose resolution does not involve a direct, personal involvement of the participant (emotionally non-engaging); ‘personal moral’ refers to moral scenario whose resolution involves a direct involvement of the participant (emotionally engaging). In the moral scenario, participants are asked to judge the appropriateness of the utilitarian response. (C) Same as (B), but for the two considered LLMs: text-davinci-002, text-davinci-003. Robot icon by Konstantin Filatov, from the Gentlecons Interface Icons collection. Licensed under CC BY.

To study how humans perceive and evaluate AI-generated moral judgments, we first collected moral dilemma responses from both human participants and language models. We gathered responses from 30 human participants and two language models, GPT-3.5 davinci-002 and davinci-003, using scenarios from Greene et al. [41]. From this, we constructed two corpora (Fig 1A): one pairing human responses with those from davinci-002 (corpus 1; dv2) and another pairing human responses with davinci-003 (corpus 2; dv3). For each corpus, we presented to 77 new participants (such that N = 153) dialogues displaying the moral scenarios and responses. Participants were asked whether the source was AI or human. Additionally, each response included a binary judgment (yes/no) and a free-text justification. By analyzing these judgments across different moral scenarios—“Non-moral,” “Impersonal moral,” and “Personal moral” (Fig 2A)—we aimed to understand how scenario type influences moral evaluations and whether AI-generated responses align with human moral reasoning.

In previous studies, decisions with utilitarian outcomes are more readily endorsed when framed in an “impersonal” manner [40,41]. For instance, participants are less keen to sacrifice one person in the “Footbridge” dilemma scenario where they themselves are the one who must push the person onto the tracks, compared to the “Trolley” dilemma, where they have to push a button to achieve the same utilitarian outcome.

Our study replicates this finding, as our human participants significantly favored impersonal moral scenarios over personal ones (Table 1A). This is consistent with previous literature using the same dataset of moral items [47–49]. Fig 2B shows the likelihood of endorsing across the three scenarios (with accompanying statistical values in Table 1). The davinci-002 and davinci-003 responses appear to follow a similar moral code: davinci-002 significantly endorses impersonal moral scenarios more than personal moral scenarios (Table 1B). Davinci-003 displayed an even greater sensitivity to this framing effect; potentially indicative of a “correct answer” bias [34] (Table 1C). This bias suggests that newer LLMs are trained and fine-tuned to generate socially accepted responses, leading to reduced diversity in their outputs. Overall, our results empirically test, and do not support, the popular lay intuition that LLMs reason in a more utilitarian fashion than humans [50]: in our data the LLMs we tested were no more utilitarian than human participants, and davinci-003 was in fact less so in personal moral scenarios.

**Table 1:** Moral Dilemma Scenarios and Endorsement Rates by Humans and LLMs. This table contains statistical values from two-tailed t-tests; each section corresponds to a section in the results. We report the following statistics for each t-test: Student’s t-value (*T* (*d f*)), the significance of the p-value (***: *p <* 0. 001, **: *p <* 0. 01, *: *p <* 0. 05, n.s.: not significant), Cohen’s d (*d*), and Bayesian factor (*BF*_10_). The source value, when numeric, refers to the corresponding corpora.

Numbering → Corpus	Feature	T(df)	*p*	*d*	*BF*_10_
***Judgment***					
**A** → humans	Impersonal vs. personal scenarios	T(30) = 2.8	*	0.69	5.02
**B** → davinci-002	Impersonal vs. personal scenarios	T(29) = 10.05	***	2.23	1.65e8
**C** → davinci-003	Impersonal vs. personal scenarios	T(29) = 49.28	***	12.11	1.09e26

### Detection accuracy across scenario types

**Fig 3:**
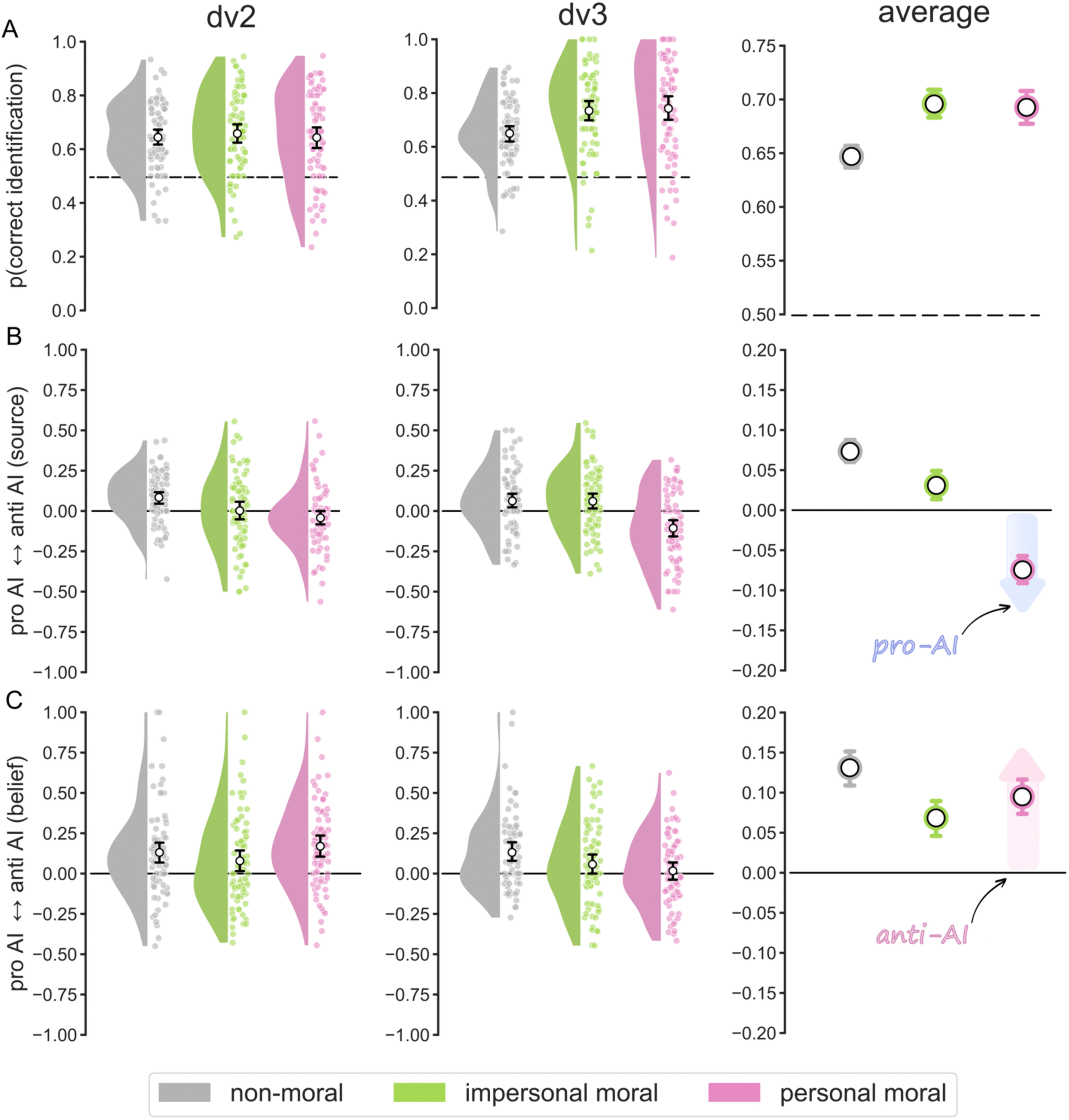
Source Detection Accuracy and Bias in Agreement Based on Actual versus Perceived Authorship. **(A)** Probability of correctly detecting the source of the judgment (p(correct identification)), as a function of the dilemma type in the dv2 corpus (leftmost column; dv2), dv3 corpus (central column; dv3), and on average (rightmost column; average). **(B)** Difference in agreement between the trials featuring human-generated items and those featuring LLM-generated items as a function of the scenario type. (**C**) Difference in agreement between the trials the participant declared as being human-generated and those declared to be LLM-generated (belief).

To assess whether participants could distinguish between human and AI-generated answers, we asked them to identify the source of each response in a Turing-test like task (Fig 1B). Importantly, participants were financially incentivized (a bonus of 5 cents per correct identification, yielding an average bonus of £1.46; see *Participants* in the Methods) for accurate performance, because their payoff was proportional to their accuracy. Across both corpora, participants’ accuracy in detecting the source varied by scenario type (Fig 3A). Overall, detection was statistically above chance but modest in absolute terms. In the dv2 corpus (humans and davinci-002 justifications), participants correctly identified the source 64% of the time (Table 2A), while accuracy increased to 71% in the dv3 corpus (humans and davinci-003), suggesting that, if anything, responses from the more advanced model were more distinguishable from human-generated justifications (Table 2B).

**Table 2:** Source Detection Accuracy and Bias in Agreement Based on Actual versus Perceived Authorship. This table contains statistical values from two-tailed t-tests; each section corresponds to a section in the results. We report the following statistics for each t-test: Student’s t-value (*T* (*d f*)), the significance of the p-value (***: *p <* 0. 001, **: *p <* 0. 01, *: *p <* 0. 05, n.s.: not significant), Cohen’s d (*d*), and Bayesian factor (*BF*_10_). The source value, when numeric, refers to the corresponding corpora.

Numbering → Corpus	Feature	T(df)	*p*	*d*	*BF*_10_
***Detection***					
**A** → dv2	Detection above chance	T(76) = 10.49	***	1.19	3.21e13
**B** → dv3	Detection above chance	T(75) = 13.88	***	1.59	2.19e19
**C** → dv3	Impersonal moral vs. non-moral	T(75) = 3.93	***	0.57	116.47
**D** → dv3	Personal moral vs. non-moral	T(75) = 4.52	***	0.56	819.58
**E** → dv2, dv3	Impersonal moral vs. non-moral	T(152) = 3.50	**	0.32	30.06
**F** → dv2, dv3	Personal moral vs. non-moral	T(152) = 3.06	**	0.28	8

Additionally, we found that the context of the scenario played a significant role in detection accuracy, though this was exclusive to the dv3 corpus. In dv3, participants were significantly more accurate at identifying the source of justifications in moral scenarios—both impersonal and personal—compared to non-moral scenarios (Table 2C, D). However, detection accuracy did not significantly differ between personal and impersonal moral scenarios themselves. Conversely, in the dv2 corpus, the type of scenario had no significant impact on participants’ ability to detect AI-generated justifications. On average, pooling both corpora, participants remained more accurate at detecting whether content was AI- or human-generated in moral scenarios compared to non-moral ones, driven primarily by the dv3 results (Table 2E, F).

### Agreement as a function of source and belief

After examining detection rates, we investigated whether agreement rates varied based on the source of the justification, and how this interacted with scenario type. Note that throughout the paper “agreement” refers to the mean of two binary items collected for each response: agreement with the judgment (yes/no decision) and agreement with the justification (free-text reasoning), coded 1/0.5/0 for full agreement, partial agreement (agreement with one but not the other), and full disagreement (see Methods, *Statistical evaluation*). Across both LLMs, we observed a significant main effect of scenario type on agreement (*F*(2*,* 152) = 8. 37*, p <* 0. 001^***^, η^2^ = 0. 09; *F*(2*,* 150) = 20. 79*, p <* 0. 001^***^, η^2^ = 0. 21)).

As shown in Row B of Fig 3, participants’ agreement with human- or LLM- responses varied by scenario type. We computed the difference in agreement rates (human minus LLM) for each scenario type. Participants significantly preferred human judgments over AI judgments for non-moral scenarios (Table 3.1.A), but showed a significant preference for AI judgments in personal moral scenarios (Table 3.1.B). No significant difference from zero was observed in impersonal moral scenarios (Table 3.1.C). These findings suggest that when faced with morally challenging scenarios, humans tend to agree more with LLM-generated justifications than with human ones. However, this pro-AI bias in personal scenarios was only significant in the dv3 corpus (Table 3.1.D) and not in the dv2 corpus (Table 3.1.E).

**Table 3:** Agreement as function of source and belief. This table contains statistical values from two-tailed t-tests; each section corresponds to a section in the results. We report the following statistics for each t-test: Student’s t-value (*T* (*d f*)), the significance of the p-value (***: *p <* 0. 001, **: *p <* 0. 01, *: *p <* 0. 05, n.s.: not significant), Cohen’s d (*d*), and Bayes factor (*BF*_10_). The source value, when numeric, refers to the corresponding corpora.

Numbering → Corpus	Feature	T(df)	*p*	*d*	*BF*_10_
**1. Agreement (source)**					
**A** → dv2, dv3	Non-moral (anti-AI)	T(152) = 5.12	***	0.58	1.35e04
**B** → dv2, dv3	Personal moral (pro-AI)	T(152) = 4.35	***	0.49	586
**C** → dv2, dv3	Impersonal moral (neutral)	T(152) = 1.73	n.s.	0.19	0.39
**D** → dv3	Personal moral (pro-AI)	T(75) = 4.15	**	0.67	232
**E** → dv2	Personal moral (pro-AI)	T(76) = 1.92	n.s.	0.31	0.72
***2. Agreement (belief)***					
**A** → dv2, dv3	Non-moral (anti-AI)	T(143) = 5.76	***	0.67	2.19e5
**B** → dv2, dv3	Impersonal moral (anti-AI)	T(143) = 2.7	*	0.31	3.08
**C** → dv2, dv3	Personal moral (anti-AI)	T(143) = 4.31	***	0.5	498.6
**D** → dv2	Personal moral (anti-AI)	T(73) = 5.16	**	0.84	7759
**E** → dv3	Personal moral (anti-AI)	T(69) = 0.65	n.s.	0.11	0.162

This pattern of agreement does not imply that participants correctly recognized the source of justifications; detection accuracy hovered around 70%. To assess whether participants’ belief about the source influenced their agreement, we re-calculated agreement differences based on the declared source (i.e., not as a function of the actual source, but as a function of the declared one) (Table 3.2).

The results are presented in Row C of Fig 3 and expose a significant anti-AI bias across all scenarios and pooled experiments (Table 3.2.A–C). Participants were more likely to agree with judgments they believed came from humans, regardless of the true source. While this anti-AI bias appeared in personal scenarios, it was only statistically significant in the dv2 corpus (Table 3.2.D), not in dv3 (Table 3.2.E).

### Humanization reduces detection and mitigates pro-AI bias

Participants’ agreement with LLM judgments varied depending on their belief about the source, especially in complex (personal) moral scenarios, suggesting that perceived authorship influences moral evaluations. Since linguistic differences could contribute to perceived authorship [14,39], we used GPT-3.5 to “humanize” davinci-002 responses in the dv2 corpus (see methods; Fig 8) by shortening them, adding typos, and mimicking human examples, which it managed to do successfully. Indeed, dv2 humanized responses showed no significant difference in length or typos (Table 4A-B). We then presented this new corpus in the form of the detection and agreement task (Fig 1) to 77 new participants.

**Table 4:** The Effect of LLM Humanization on Detection Rates and Agreement Differentials. This table contains statistical values from two-tailed t-tests; each section corresponds to a section in the results. We report the following statistics for each t-test: Student’s t-value (*T* (*d f*)), the significance of the p-value (***: *p <* 0. 001, **: *p <* 0. 01, *: *p <* 0. 05, n.s.: not significant), Cohen’s d (*d*), and Bayesian factor (*BF*_10_). The source value, when numeric, refers to the corresponding corpora.

Numbering → Corpus	Feature	T(df)	*p*	*D*	*BF*_10_
***Corpus 3: dv2 humanized***					
**A** → Davinci-002-h, humans	Length	T(2982) = 1.51	**ns**	0.118	0.05
**B** → Davinci-002-h, humans	Typos	T(3486) = 0.30	**ns**	0.04	0.01
**C** → dv2h	Detection above chance	T(76) = 8.24	***	0.93	2.3e09
**D** → dv2h	dv2h is source neutral	T(76) = 0.94	**ns**	0.1	0.19
**E** → dv2h,dv2	anti-AI bias reduced; dv2 vs dv2h	T(144) = 1.97	ns	0.32	1.05
**F** → dv2h	anti-AI bias above chance (belief)	T(75) = 3.57	*	0.40	37.82

**Fig 4:**
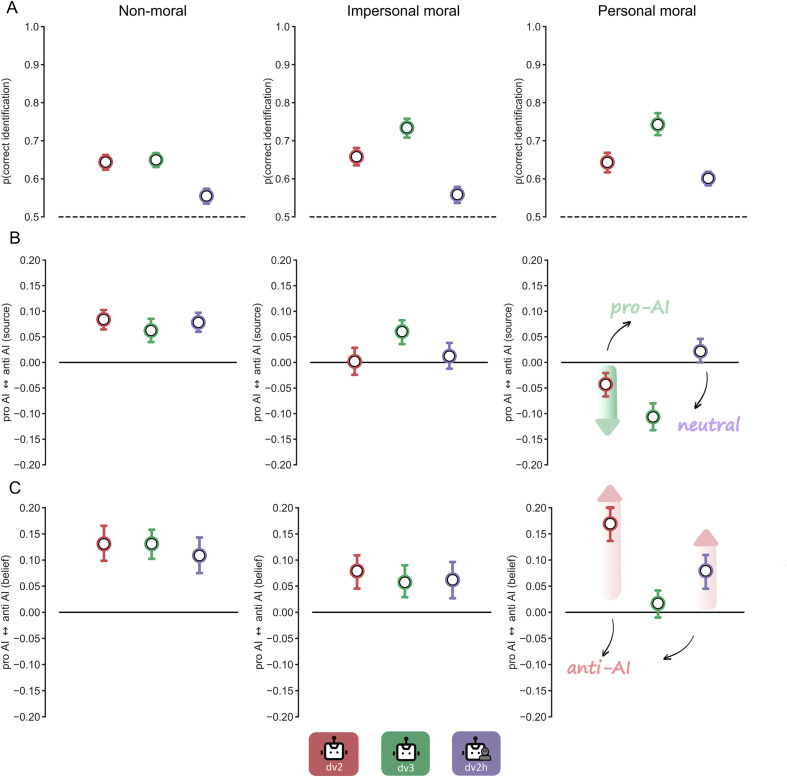
The Effect of LLM Humanization on Detection Rates and Agreement Differentials. **(A)** Correct identification rate in the detection task across different corpora: dv2 corpus (dv2, red), dv3 corpus (dv3, green), and dv2 humanized corpus (dv2h, purple). Columns represent different scenario types: Non-moral (left), Impersonal moral (center), and Personal moral (right). Dots represent mean values, and error bars indicate the standard error of the mean. **(B)** Actual source-oriented agreement differential, representing the difference in agreement between trials featuring human-generated items and those featuring LLM-generated items. **(C)** Declared source-oriented (belief) agreement differential, reflecting the difference in agreement between trials participants believed were human-generated versus those they believed were LLM-generated. Robot icon by Konstantin Filatov, from the Gentlecons Interface Icons collection. Licensed under CC BY.

**Fig 5:**
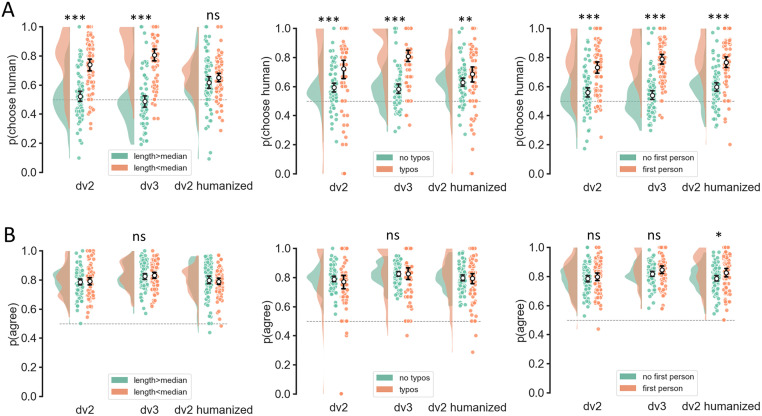
The Influence of Surface Linguistic Features on Source Identification and Participant Agreement. (A) Probability of choosing ‘human’ in the detection task as a function of different linguistic features. Items are split as a function of their length (leftmost column), the presence or not of typos (central column), and the utilization of first-person marker (rightmost column). (B) Probability of agreeing with justification as a function of linguistic features.

**Fig 6:**
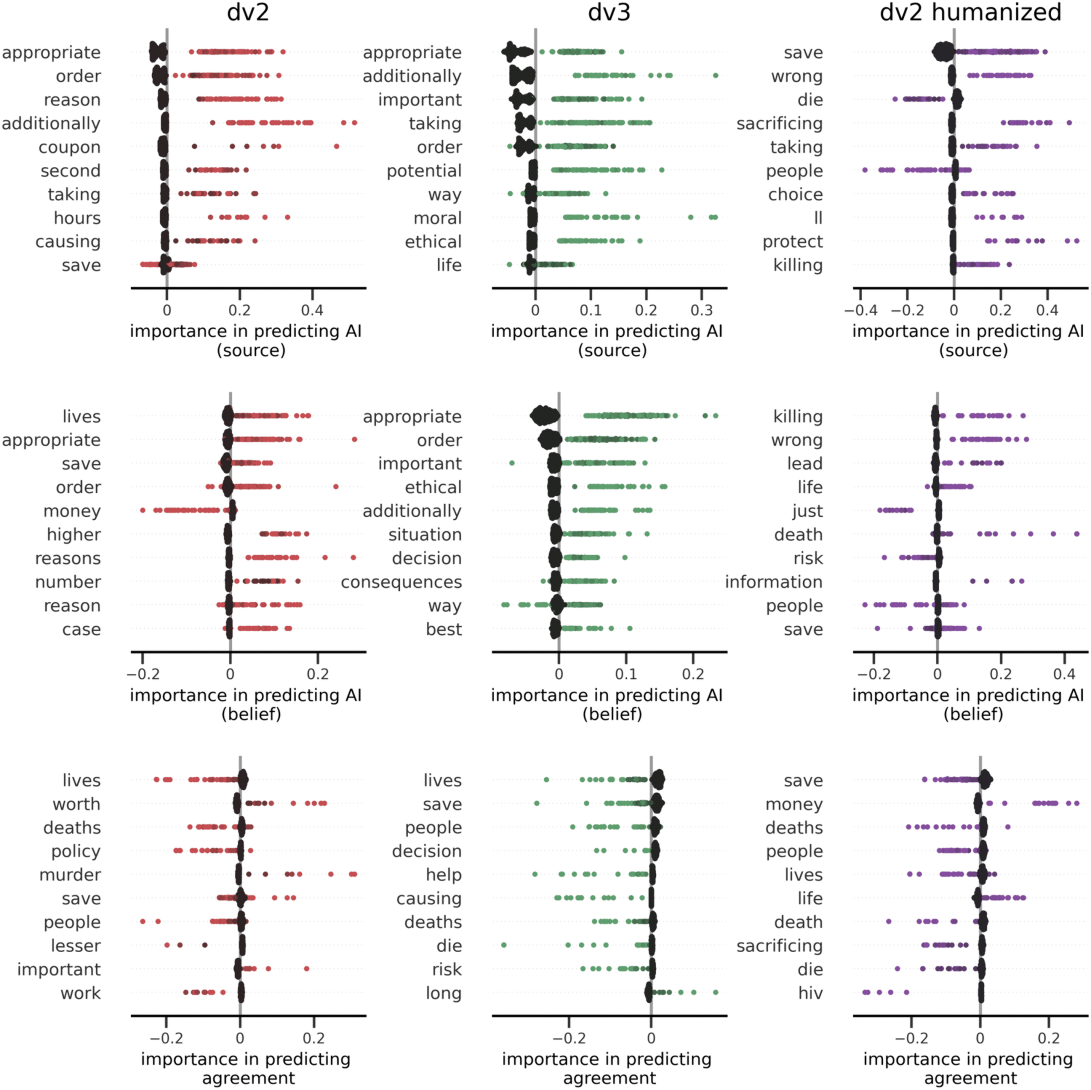
SHAP Analysis Identifying Semantic Features that Predict Source, Belief, and Agreement. The model predicted three variables (rows: source, belief, agreement) for each corpus (columns; dv2, dv3, dv2 humanized). Each point represents a data instance (i.e., a trial in the detection and agreement task), with the x-axis showing the SHAP value, which reflects the importance of a word in influencing the prediction (i.e., an identification of AI/human generated judgments, or an agreement with the text). The color represents the feature value, which in this case is the word’s frequency. Black dots indicate a low frequency of the word for a given sample of moral judgments, while colored dots signify a higher frequency. Thus, the feature value (color) goes from black (low word occurrence) to brighter colors for words that appear more frequently. Features are ordered by average absolute SHAP value, highlighting their relative importance. Features with higher SHAP values have a larger influence on the model’s output, with the most important features appearing at the top. We only display the 10 most important features.

**Fig 7:**
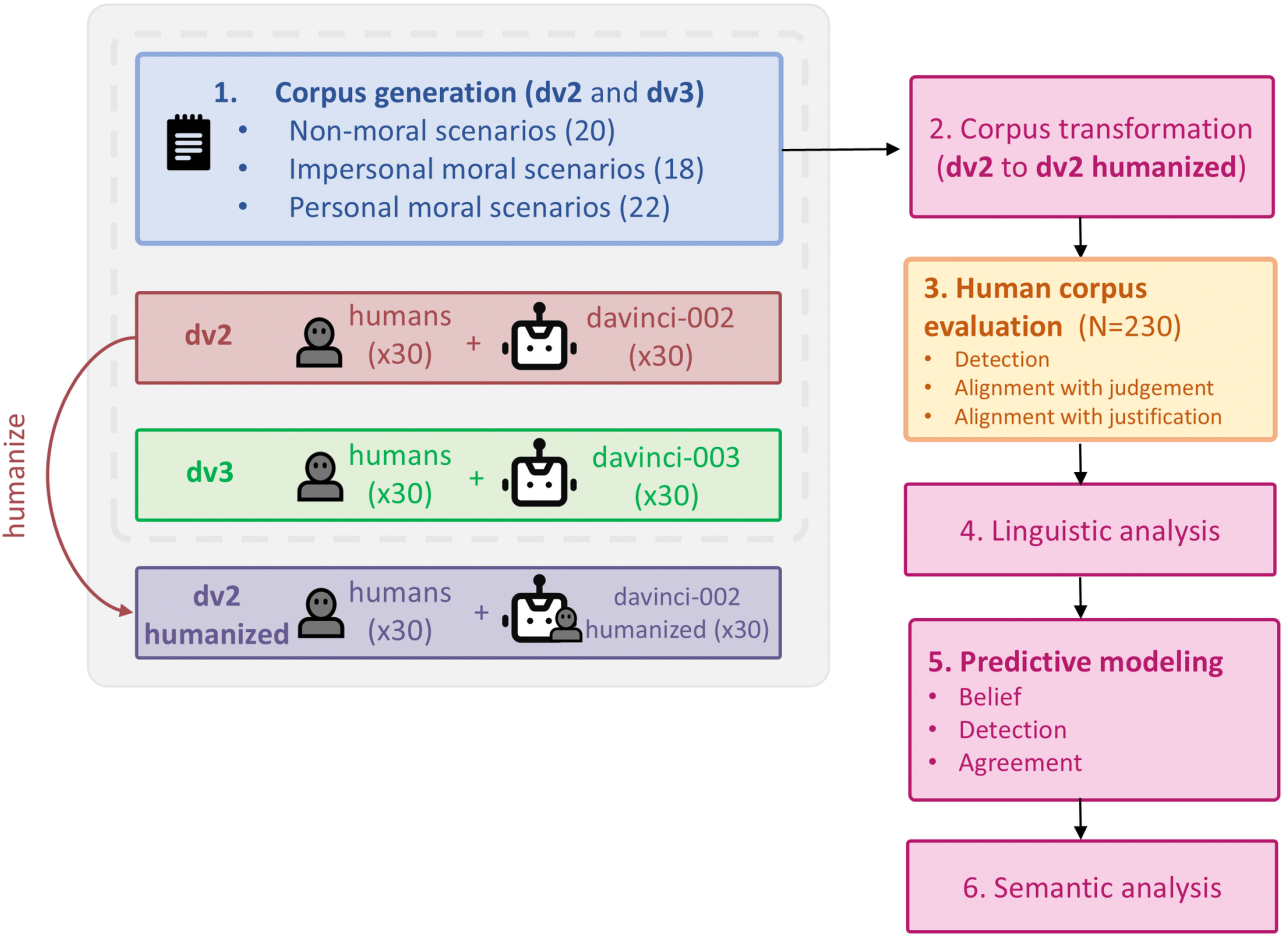
Comprehensive Overview of the Experimental Design and Analytical Workflow. A diagram showing the experimental design. The blue and orange boxes (Steps 1 and 3) correspond to respectively Corpus generation and Detection and Agreement experiments (see Fig 1). The purple boxes (Steps 2, 4, 5, and 6) denote quantitative, statistical, and computational methods. The output of the experiment is an analysis on three corpora of human- and LLM- generated responses to various scenarios. Robot icon by Konstantin Filatov, from the Gentlecons Interface Icons collection. Licensed under CC BY.

**Fig 8:**
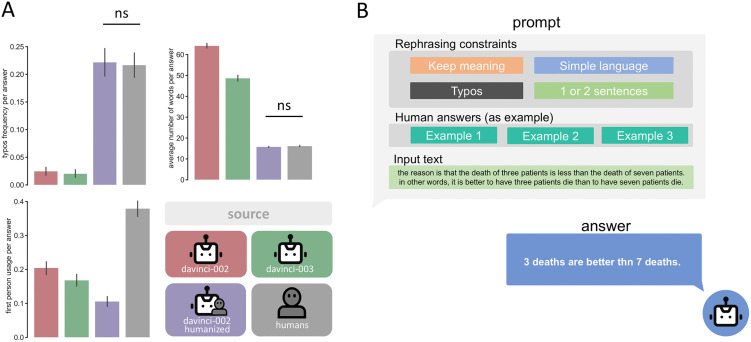
Linguistic Distinctions and the Prompting Strategy for Humanizing LLM Outputs. (A) Identified linguistic features which have been found to be different between human- and LLM-generated responses. (dv2: text-davinci-002; dv3: text-davinci-003, dv2 humanized: humanized dv2). (B) Schematized prompting strategy to generate the humanized LLM response, by reducing size and including typos. Robot icon by Konstantin Filatov, from the Gentlecons Interface Icons collection. Licensed under CC BY.

Comparing the results from dv2, dv3 and dv2 humanized (dv2h), humanization was successful in significantly reducing the detection rate in non-moral (F(2, 227) = 12.73, p < 0.001***, η^2^ = *0.10*) impersonal moral (F(2, 227) = 24.06, p < 0.001***, η^2^ = *0.17*), and personal moral scenarios (F(2, 227) = 14.43, p < 0.001***, η^2^ = *0.11*). The latter dropped below 60% but still remained significantly above 50% (Table 4C) (Fig 4A).

When focusing on the actual source, the difference in agreement (i.e., the bias) between human and machine items remained unchanged in non-moral (*F(2, 227) = 0.33, p = 0.721, η*^2^ = *0.003*) and impersonal moral scenarios (*F(2, 227) = 1.51, p = 0.222, η*^2^ = *0.01*). However, when in personal moral scenarios participants tend toward a pro-AI bias in dv2 and dv3 corpora, humanization led to a more neutral stance (F(2, 227) = 7.32, p < 0.001***, η^2^ = *0.06*) (Fig 4B) (Table 4D), which probably indicates that the acceptability of moral judgments is influenced by their linguistic quality. When considering participants’ belief about the source, an anti-AI bias remained (i.e., they agree less when they think the content is AI-generated) in non-moral (*F(2, 227) = 0.16, p = 0.853, η*^2^ = *0.001*) and impersonal moral scenarios (*F(2, 227) = 0.12, p = 0.887, η*^2^ = *0.001*). Concerning personal moral dilemmas, humanizing did not significantly affect the anti-AI bias compared to dv2 (Table 4E) and remained significantly higher than zero (Fig 4C; Table 4F). In summary, ‘humanizing’ LLM responses by shortening them and introducing typos, successfully managed to reduce the detection of their response, but had no substantial effect on the belief-based anti-AI bias.

### Linguistic cues shape both detection and agreement

So far, we have found that participants are weakly able to detect LLM-generated moral justifications, and that this detection varies across scenario type. Is it the case that linguistic features are driving this detection ability? In Fig 5, we quantify the influence of linguistic features – length, typos, and first-person usage (columns 1–3) – and plot their effects on the probability of detection (row A) and alignment (row B). In dv2 and dv3, we see that length, typos, and first-person usage all significantly affect detection rates (Table 5A-F), suggesting that participants relied on such linguistic cues to improve their source identification.

**Table 5:** The Influence of Surface Linguistic Features on Source Identification and Participant Agreement. This table contains statistical values from two-tailed t-tests; each section corresponds to a section in the results. We report the following statistics for each t-test: Student’s t-value (*T* (*d f*)), the significance of the p-value (***: *p <* 0. 001, **: *p <* 0. 01, *: *p <* 0. 05, n.s.: not significant), Cohen’s d (*d*), and Bayesian factor (*BF*_10_). The source value, when numeric, refers to the corresponding corpora.

Numbering → Corpus	Feature	T(df)	*p*	*d*	*BF*_10_
***Linguistic analysis***					
**A** → dv2	Length	T(76) = 8.00	***	1.25	3.44e9
**B** → dv3	Length	T(75) = 10.92	***	1.8	1.139e15
**C** → dv2	Typos	T(75) = 4.02	***	0.58	149.147
**D** → dv3	Typos	T(75) = 10.33	***	1.41	1.391e13
**E** → dv2	First-person usage	T(76) = 7.31	***	1.04	4.423e07
**F** → dv3	First-person usage	T(75) = 13.28	***	1.68	2.143e18
**G** → dv2h	Length	T(76) = 0.16	ns	0.01	0.127
**H** → dv2h	Typos	T(76) = 3.09	**	0.41	9.94
**I** → dv2h	First-person usage	T(76) = 8.72	***	1.09	1.786e10

As expected, in the dv2 humanized corpus (where we targeted reducing these differences, except for first person use), these effects are greatly diminished. Indeed, the length of the text is no longer a significant predictor of human authorship (Table 5G). Interestingly, even though there were no significant differences in typos between human- and (humanized) AI-generated texts in the actual dataset, participants still used typos as a cue for detection, albeit to a lesser extent (Table 5H). Finally, first-person usage still significantly predicts human detection (Table 5I). This suggests that humans can pick up on subtle linguistic cues, such as first-person pronoun usage, even in the absence of more explicit markers like text length. It also indicates that some systematic heuristics, such as the presence of typos, may play a role in these detection heuristics [14].

We also test whether these three linguistic factors predicted agreement. The only significant effect was found for first-person usage in the humanized condition. Since linguistic features did not consistently predict agreement, this suggests that deeper conceptual content, semantic factors, and subjective preferences likely play a larger role in shaping alignment.

### Semantic cues such as cost-benefit phrasing reveal AI authorship and drive moral disagreement

To identify the semantic features that influence human judgments in detection and agreement tasks, we trained a random forest classifier using data from the dv2, dv3, and dv2 humanized corpora. The classifier was trained to predict three variables—specifically, whether the text actually originated from AI (Fig 6; first row), whether participants identified a text as AI-generated (Fig 6; second row), and, finally, whether they endorsed the moral justification provided (Fig 6; third row). To interpret the model’s predictions and understand the contribution of each semantic feature to these predictions, we applied SHAP (SHapley Additive exPlanations) analysis.

In dv2 and dv3, SHAP analysis showed that the word “appropriate” was a strong indicator of

AI-generated content (Fig 6; first row). Indeed, LLM responses often reworded the question. For example, if the question was worded like, “Is it appropriate to do X to save Y?” the LLM would begin its response with, “Yes, it is appropriate because...” Participants picked up on this, often identifying justifications containing the term “appropriate” as AI-generated (Fig 6; second row). In particular, davinci-003 appeared more pedantic and repetitive, which may contribute to its high detection rate. The following are examples of davinci-003-generated justifications that the classifier model was able to correctly identify with high probability. See the following example:

*“it is not* ***appropriate*** *to leave this man by the side of the road in order to preserve your leather upholstery because human life is more important than material possessions. taking the time to help this man in need is the right thing to do and it is your moral responsibility to prioritize his health and safety over your car’s upholstery. it may be inconvenient to have to clean up the mess and deal with the ruined upholstery, but it is a small price to pay for saving someone’s life.”*— davinci-003

Words like “additionally” and “reason”, which imply logical reasoning, were recognized by our classifier model as markers of AI-generated judgments (Fig 6; first row). These words were also predictive of human detection of AI (Fig 6; second row). This suggests that both the classifier and participants detected formal reasoning language as a distinctive feature of AI-produced judgments. The following example shows a correctly predicted dv2 instance demonstrating logical reasoning:

*“killing someone, even your own child, is never an* ***appropriate*** *solution. human life is inherently valuable, and no one should be asked to make the impossible decision of sacrificing a life for the sake of others.* ***therefore****, it is not appropriate for you to kill your oldest son in order to save your husband and [other] children.”*— davinci-002

Words tied to cost/benefit calculations (e.g., “lives,” “save,” “deaths”) were predictive of disagreement with justifications (Fig 6; third row). These words often appear in justifications to personal moral scenarios, which was the category of scenarios receiving the least endorsement by both humans and LLMs (Fig 2). For example, one such scenario asks whether it is appropriate for a doctor to sacrifice one patient to save five others. Participants often disagreed with the utilitarian outcomes in this case, highlighting the moral conflict. In contrast, the presence of terms like “murder” predicted agreement, often used in moral statements like “the murder of innocents is always wrong”, aligning with deontological principles [48].

The predictive terms varied in the dv2 humanized corpus (where GPT-3.5 was prompted to imitate human responses), both in predicting the source of the judgments (Fig 6; first row) and participants’ beliefs (Fig 6; second row). Words like “save,” “sacrificing,” “choice,” and “killing” (likely associated with cost-benefit calculations), frequently appeared in AI-generated content. Participants recognized these cues as AI-generated even in the absence of low-level cues (e.g., text length and typos). This reflects large language models’ tendency toward cost-benefit reasoning, which participants likely used as a heuristic to detect AI-generated responses.

Agreement in the dv2 humanized corpus (Fig 6; third row) followed similar patterns as dv2 and dv3: words evoking personal moral scenarios, such as “deaths,” ‘lives,” and “sacrificing,” often led to participant rejection. These terms likely reflect emotionally charged conflicts between utilitarian outcomes and necessary actions.

## Discussion

### Summary

In our study, we aimed to study how humans perceive and discriminate AI-generated moral reasoning, in experimental corpora generated by administering a well-established moral psychology task designed to elicit contrasting moral preference across a diversity of scenarios [40,41].

In the corpora generation experiments, we replicated the finding that human preferences are scenario-dependent; they endorse more actions that are morally irrelevant (i.e., non-moral scenarios) and less readily endorse actions that involve complex trade-off between deontological and utilitarian principles (personal and impersonal moral dilemma). We administered the same task to LLMs [46,51,52] and found overall alignment in the direction of the effects, but also fine-grained misalignment between human- and LLM-generated endorsement rates, especially across scenario type [30,34,53].

In our main experiments, participants evaluated responses from our experimentally generated corpora. Overall, participants were only moderately successful at distinguishing between the moral justifications generated by humans and LLMs, and the context of the scenario was important (morally charged justifications were generally detected at a higher rate). For justifications on relatively trivial matters, participants generally showed greater agreement with human justifications. In contrast, participants preferred AI-generated responses for complex moral scenarios. This pro-AI bias (defined as the differential of agreement with human vs. AI-generated texts) for complex moral scenarios was not explicitly recognized by participants. Rather, it coexisted with a pervasive belief-based anti-AI bias, where participants expressed higher agreement with justifications they believed came from humans, regardless of the actual source. However, it’s worth noting that ‘humanizing’ the corpus at a low linguistic level had a robust effect on detection but did not influence the belief-based anti-AI bias.

Finally, while low level linguistic features (length of the justification, typos and use of first person) were shown to significantly contribute to detection, they had no detectable impact on agreement. SHAP-based analysis further revealed that higher-level semantic content played a more substantial role in shaping participants’ agreement.

### Scenario complexity modulates preference for AI judgments

Participants exhibited a significant preference for AI-generated judgments in personal moral scenarios, which typically involve more deliberation and evoke stronger emotional responses (e.g., pushing one person off a bridge to save five). In contrast, for less emotionally engaging, impersonal scenarios (e.g., diverting a runaway boxcar), participants presented balanced preferences. In personal moral scenarios, where participants perceive a higher level of conflict, dual-process accounts of moral cognition predict that it can be resolved by deliberate reasoning [41]. This may explain the preference for AI judgments, which might be perceived as more reasoned compared to human ones. However, this framework is debated, with some arguing that the distinction between intuitive and rational processes is not always clear-cut [54].

### Belief-driven anti-AI bias overrides actual source

The second major finding, concerning the influence of participants’ beliefs on their agreement with moral judgments, reveals a complex interaction between belief-based and source-based biases. Participants more often expressed disagreement with moral justifications they believed as AI-generated, irrespective of their actual source (anti-AI bias). This was true regardless of the scenario under consideration. Additionally, humanizing davinci-002 responses helped mitigate the pro-AI stance in complex moral scenarios, while participants still tend to prefer what is perceived as human. This result is further consistent with the idea that human favoritism stems from labeling an output as human-generated, rather than from the actual content itself [55]. Similar patterns have been observed in other domains, such as game theory, where revealing the content source negates the benefits of AI-generated outputs [56]. These findings align with previous research, suggesting that humans do not exhibit a simple aversion to AI [16,27–29,37,57].

### Preferred but unattributed: the LLM authorship paradox

In line with recent work, our findings uncover a persistent dissociation between the detectability and the perceived quality of LLMs’ generated moral justifications. While participants showed above-chance accuracy in identifying AI content in our study, consistent with the distinguishability found in other models, we also confirm that LLMs are successfully passing a comparative Turing test, aimed, not only at assessing detection but also relative valuation of human and AI-generated moral justifications [58]. Specifically, Aharoni et al. [36] and Dillion et al. [22] reported LLM-generated responses as being rated superior in dimensions like “intelligence” and “morality”. Similarly, our participants displayed a pro-AI bias for justifications involving complex (personal) moral scenarios. However, our study introduces a critical distinction by demonstrating that the preference toward LLM-generated responses is accompanied by the belief that they are not the actual source of justification. In other terms, while the objective quality of LLMs in generating high-quality moral discourse is recognized, this is accompanied by a tendency to deny them authorship of the material.

### In-group/out-group bias as an explanatory mechanism

One explanation for this misattribution is an ingroup/outgroup bias [59], potentially driven by the cognitive dissonance of preferring an AI’s judgment [60–62]. This bias, recently evidenced as human favoritism against AI [27] may not require a deep representation of AI as a social agent. Based on the Minimal Group Paradigm, the mere categorization of ‘human’ (ingroup) versus ‘AI’ (outgroup) can be sufficient to elicit favoritism. This ‘us vs. them’ dynamic is further activated by prevalent cultural narratives framing AI as a ‘Pandora’s Box’ threat, focusing on human replacement [63].

This effect is likely amplified in moral contexts. Ingroup favoritism intensifies when core values are at stake [64], leading to biased rationalizations [65]. The rejection of AI’s moral agency [29] may be a key catalyst. As Bigman and Gray [29] posit, AI may be categorized as an outgroup precisely *because* it is perceived as a non-moral agent, a social being that is fundamentally different from humans. In our experiments, given that textual and linguistic features were the only cues available, they were the likely determinants of this ingroup identification.

### Surface linguistic features drive detection alone

We conducted several analyses to understand how the different sources (humans, davinci-002, davinci-003, and davinci-002-humanized) varied from each other and how specific features influenced detection and agreement. First, we examined linguistic features such as justification length (LLM generated longer responses) and the presence of typos (LLMs rarely made errors). As expected, these features significantly aided human detection, based on the assumption that LLMs tend to produce longer, typo-free responses [23]. Reducing typos and shortening responses substantially lowered detection rates when evaluating dv2 humanized corpus (human vs. davinci-002 humanized). However, detection accuracy remained above chance, even after these modifications. Consistent with previous findings, humans demonstrated a strong tendency to use first-person discourse more frequently than LLMs [14]. Notably, neither the textual features (length, typos) nor syntactic features (first-person discourse) were linked to agreement judgments, suggesting that human moral reasoning is unaffected by changes in these basic linguistic aspects.

### Semantic reasoning markers shape both detection and agreement

To further explore these findings, we applied SHAP interpretations from a classifier model to predict the source of the text. The analysis revealed that terms indicating structured reasoning (e.g., “additionally,” “reason”) were strong predictors of AI-generated content, recognized both by the model and by participants.

Interestingly, while detection and source prediction overlapped in some cases, they diverged in the dv2 humanized corpus (where GPT-3.5 mimicked human responses). In these cases, humanized responses removed many typical cues. However, semantic patterns revealed that formal reasoning in the predictive tokens was still identifiable, even when formal textual features were diminished. Participants relied on these patterns to achieve modest but significantly above-chance detection rates.

Semantic patterns also revealed that cost/benefit calculation terms (e.g., “lives,” “save”) were associated with disagreement particularly in personal moral scenarios like sacrificing one person for many. This aligns with existing literature, as people tend to expect AI to engage in formal reasoning [50]. Importantly, in our results LLMs were not more utilitarian (if anything, davinci-003 was adopting deontological stances in personal moral scenarios more than humans), yet the cost-benefit calculation terms that it typically relied on were used as a cue by participants. Cost-benefit vocabulary functions as two distinct cues: (i) a marker of utilitarian reasoning within the content itself, and (ii), independently, a folk-psychological cue to AI authorship. Empirically in our data these came apart: the LLMs we tested were not more utilitarian than humans, yet participants still used this vocabulary to infer AI authorship. Thus, while basic textual and syntactic features affected detection, semantic elements tied to moral reasoning played a key but nuanced role in agreement with judgments.

### Limitations and moral-alignment caveats

A key limitation of our study is that the findings may be specific to GPT-3.5 and might not generalize to other models. The behavior of LLMs can vary based on their architecture and the specific version used, and the quality of LLM-generated moral justifications has increased substantially in more recent model families (e.g., GPT-4, GPT-5 and their successors). Our results should therefore be read as a snapshot tied to this generation of models. In particular, detection accuracy, the magnitude of the pro-AI bias in personal scenarios, and the persistence of the belief-driven anti-AI bias are all empirical questions that should be re-tested with more recent systems. We view the belief-based dissociation as the finding most likely to generalize, since it is grounded in an ingroup/outgroup dynamic rather than in any specific property of the model; but this prediction itself requires further testing. However, the alignment results from the ‘corpus’ generating experiments were not central to our main claim regarding how LLM judgments are detected and evaluated. Another limitation of our study is that we do not explicitly classify responses as utilitarian or deontological, which makes it challenging to disentangle the complex interplay between moral alignment, language use, and AI detection. For instance, we observe that pro-AI biases (source attribution) in personal moral scenarios specifically emerge with davinci-003, while anti-AI biases (belief attribution) appear with davinci-002. Moreover, with davinci-003 exhibiting more deontological tendencies [34] and davinci-002 being more utilitarian (Fig 2), future studies should further investigate how moral alignment interacts with AI detection and agreement in shaping participants’ responses.

### Residual detectability despite humanization

Additionally, participants’ imperfect detection of AI-generated judgments may stem from linguistic factors, such as subtle differences in phrasing or style. Despite efforts to humanize LLM responses in corpora 3, participants still detected LLM-generated judgments above chance. As shown in Fig 5, they relied on (notably) first-person cues as a decision heuristic [14,39]. Moreover, with careful prompting, AI-generated judgments can become even harder to detect, and in some cases, LLMs have been rated as more human-like or empathetic than actual human responses [14,24,66].

### Dissociating moral agency from moral judgment quality

Our findings reveal a potential dissociation between participants’ attribution of moral agency to AI systems and their evaluation of AI-generated moral judgments. While participants might reject the notion that AIs can act as true moral agents, as supported by previous research [29] they nonetheless find AI-generated judgments persuasive, especially in complex scenarios that challenge their own moral intuitions. This asymmetry suggests a dissociation between attributed moral agency and perceived judgment quality, where participants maintain an anti-AI bias concerning moral agency but exhibit a pro-AI bias when practically evaluating the quality of moral judgments. However, when humanizing GPT-3.5 outputs, and therefore making AI and humans’ outputs harder to distinguish, this preference for actual AI-content disappeared.

### LLMs as persuasive but unacknowledged moral advisors

Our study further demonstrates that large language models (LLMs) exhibit human-like reasoning that can deviate from utilitarian standards depending on how the moral scenario is framed [67]. These deviations mirror those observed in humans, and, in the case of GPT-3.5, may even be more pronounced. Participants frequently struggled to distinguish between human and AI- generated moral justifications, raising concerns about the potential for LLMs to mislead users. Beyond producing outputs that are difficult to detect, LLMs can be further optimized to appear even more human-like, increasing this risk.

Moreover, human agreement with LLM justifications was influenced by the nature of the moral scenario, with participants showing a stronger preference for AI judgments in more complex scenarios. This suggests a potential role for LLMs as advisors or mediators in moral decision-making. However, this pro-AI bias emerged largely without participants’ awareness; agreement was higher for justifications believed to come from humans, regardless of the actual source. This gap between perceived and actual source highlights how AI aversion or human favoritism may complicate the integration of LLMs into morally sensitive domains.

## Methods

Our design (Fig 7) involved the following steps. None of the studies were pre-registered.

**Step 1: Corpus generation.** First, we create two corpora of responses to scenarios of various types: non-moral, impersonal moral, or personal moral. For each scenario in corpus 1 (dv2), 30 human participants and 30 API calls of GPT 3.5 text-davinci-002 provide a *response*, which includes a *judgment* (yes/no) and a *justification* (free text). Corpus 2 (dv3) uses GPT 3.5 text-davinci-003 instead of text-davinci-002. These two corpora provide the raw material for the detection task, but first we address a potential linguistic confound.

**Step 2: Corpus transformation.** Because human- and LLM-generated justifications may have linguistic differences (such as typos, or response length), we create corpus 3 (dv2 humanized), which “humanizes” the davinci-002 responses from corpus 1 by adding typos or shortening the text. With all three corpora in place, we can now turn to the main behavioral experiment.

**Step 3: Corpus evaluation.** Next, we have human participants evaluate each response from the 3 corpora. For each evaluation, they answer 1) whether they think the text was human- or LLM-generated, 2) whether they agree with the *judgment*, and 3) whether they agree with the *justification*. The behavioral data alone does not reveal *why* detection and agreement vary across scenarios and sources; the next three steps use statistical and computational analyses to isolate the textual signals that drive these effects.

**Step 4: Linguistic analysis.** We first perform a linguistic analysis on potential surface-level differences between human- and LLM-generated text, such as length, typos, and use of first-person pronouns. Beyond these surface features, we also ask whether deeper content patterns in the justifications carry predictive signal.

**Step 5: Predictive modeling.** We use NLP techniques to evaluate whether the responses in the corpora have predictive signals on our outcomes of interest. We build models to predict 1) the true source of the text, 2) the human participants’ belief of the source of the text, 3) whether the human rater’s belief was correct, and 4) whether the human agrees with the justification. To move from predictive accuracy to interpretability, we finally identify which specific tokens drive these predictions.

**Step 6: Semantic analysis.** Finally, we use interpretability methods to identify *which specific tokens* in the justification text have predictive power on detection and agreement.

### Corpus generation

In the preliminary item-generating stage of this study (Fig 1A; corpus generation experiments), we presented 60 moral scenarios from Greene et al. [41] to participants. In addition to having been actively used to the point of becoming a well-established benchmark in the field of moral psychology, they are still actively used by other teams [68]. There were three categories of these scenarios:

- **Non-moral**: Scenarios that do not engage complex moral reasoning, e.g., “Is it appropriate to wait for a promotion before buying a new computer, versus buying it immediately?”
- **Impersonal moral:** Moral scenarios involving indirect intervention, e.g., “Is it appropriate to turn a runaway boxcar away from five people but towards one person?”
- **Personal moral:** Emotionally salient scenarios that are more engaging, e.g., “Is it appropriate to push one person off a bridge to stop a runaway boxcar from hitting five people?” These scenarios are similar to impersonal moral scenarios but involve a sense of personal agency.

Participants provided a yes/no decision and a free-text justification for each scenario. Each corpus contained 3600 responses, derived from 60 scenarios with 30 human responses and 30 LLM responses per scenario.

The temperature of the LLM calls was set to 0.7, to maintain a form of inter-prompt variability similar to what is observed in humans. After removing ambiguous answers, defined as responses where the binary yes/no judgment could not be determined from the free-text justification (e.g., “maybe,” “it depends,” or replies that argued both sides without committing to a judgment), we created two corpora:

- **Corpus 1 (dv2)**: Initially contains 1800 responses (30 agents multiplied by 60 moral items) from text-davinci-002 batch of experiments and 1800 responses from humans (see Fig 1A; corpus generation). After manually cleaning up the dataset by removing ambiguous answers, 3542 answers were retained.
- **Corpus 2 (dv3)**: Initially contains 1800 responses (30 agents multiplied by 60 moral items) from text-davinci-003 batch of experiments and 1800 responses from humans (see Fig 1A; corpus generation). After manually cleaning up the dataset by removing ambiguous answers, 3420 answers were retained.

### Corpus transformation

To mitigate any potential linguistic differences, we used GPT-3.5 to ”humanize” the LLMs’ responses in corpus 1 by shortening them, introducing a few typos, and providing human examples to imitate; this process is shown in Fig 8. Below is the prompt we submitted to GPT-3.5 (davinci-003) that was used to transform each previously generated moral justification from davinci-002:

*Modify the input text following these instructions:*
*- keep the same meaning and ideas*
*- use simple language, avoid using abstract concepts*
*- summarize the input text into 1 sentence or 2 very short sentences (keep it under 120 characters)*
*- occasionally make spelling mistakes (e.g., ““teh”” instead of ““the””) but not too often*
*- take inspiration (in terms of phrasing and length, not the content) from these examples from real humans:*
*example 1: {randomly sampled human justification #1}*
*example 2: {randomly sampled human justification #2}*
*example 3 {randomly sampled human justification #3}*
*input text: {AI-generated justification}*
*output text:*

Of note, the strict application of this prompt revealed that many typos were introduced, such that we later used language-tool-python (2.9.4) to correct some of them (~20%). This allowed us to reach a similar typo rate as the one shown in the human dataset.

Our resulting dv2 humanized dataset showed no significant difference in length or number of typos from the human dataset, as evidenced by statistical t-tests. This resulting dataset of human and davinci-002 humanized responses is **corpus 3 (dv2 humanized)**.

### Corpus evaluation

In the main stage of the study (Fig 1B), we presented the responses generated in the first stage to new human participants (N = 230). Each corpus (dv2, dv3 or dv2 humanized) was first randomized, then divided into chunks, whose size was determined in order to ensure that task duration was kept below 45 minutes. 77 participants evaluated 46 responses in corpus 1 (total size = 3542), 76 participants evaluated 45 responses in corpus 2 (total size = 3420), and 77 participants evaluated 46 items in corpus 3 (total size = 3542). On average, participants encountered 50% ± 7% of AI-generated items.

For each response, participants had to answer three binary questions:

1. **Detection:** Whether they think these answers were given by humans or AI (human/AI)
2. **Agreement with the binary judgment:** Whether they agree with the binary moral judgment (yes/no)
3. **Agreement with the justification:** Whether they agree with the provided justification (yes/no)

#### Participants.

The research was conducted in accordance with the principles and guidelines for experiments involving human participants as outlined in the Declaration of Helsinki (1964, revised in 2013). The study received approval from Paris School of Economics ethical committee (2024−007). Informed consent was obtained from all participants prior to their involvement in each experiment. The data collection period spanned from February 27, 2024, to May 25, 2024.

The corpus generation experiment involved 30 human participants (15 females, mean age = 34 *±* 10. 26) (Fig 1A). The detection and agreement experiments involved 230 participants (113 females; mean age = 35. 57 *±* 11. 71) (Fig 1B). They were recruited through Prolific with the screening requirement that they were fluent in English and currently located in either the United Kingdom (UK) or the United States (US). In the item generation phase, 87% of the participants were from the UK and 13% were from the US. For the main evaluation, 90% of the participants were UK-based and 10% were US-based. Instructions were fully transparent, informing participants that they are expected to give meta-judgments on both human and AI-generated responses. In addition to the base rate, participants were incentivized in Turing test questions with a bonus of 5 cents for each correct identification (AI/human). The average final bonus was *£*1. 46 *±* 0. 28, which was significantly higher than what they would have received on average for making random choices (*T* (229) = 17. 28, *p* *<* 0. 001^***^, *d* = 1. 14*, BF*_10_ = 9. 768 × 10^39^). The experiments lasted, on average, 45 minutes.

#### Statistical evaluation.

The main outcome measures from the corpus evaluation steps are the binary responses to the three questions: detection (human- or LLM- generated), agreement with judgment (yes/no), and agreement with justification (yes/no). The responses to the ’detection’ question were transformed into an accuracy measure. The two agreement answers were averaged, such that a full agreement was coded as 1, a partial agreement as 0.5, and a complete disagreement as 0.

All inferential tests were conducted using Python 3.9 and the Pingouin 0.5.4 library. Two-tailed t-tests were performed throughout. For multiple comparisons, we used the pairwise_ttests function with Bonferroni corrections systematically applied. Single t-tests were performed using the ttest function. We report the following statistics for each t-test: Student’s t-value (*T*(*df*)), p-value significance (*p*), Cohen’s d (*d*), and Bayesian factor (*BF*_10_).

The anova function was used for comparing independent samples (one-way or two-way ANOVA), and the rm_anova function was used for repeated measures ANOVA (one-way ANOVA with repeated measures). For ANOVAs, we report the F-statistic (*F*(*df*)), p-value (*p*), and partial eta-squared (*η*^2^).

### Linguistic evaluation

We performed the corpus transformation step because we anticipated that linguistic differences between human- and LLM-generated text could be contributing to detection and alignment. Specifically, we hypothesize that 1. humans generate shorter justifications in length, 2. humans create more typing errors, and 3. humans tend to write more often in the first-person. We perform a statistical analysis to evaluate these differences.

### Predictive modeling

Next, we wanted to understand whether state-of-the-art models could glean predictive signals for the outcomes of interest within the justification text.

**Pre-processing** Lexical and semantic analyses were performed using NLTK tokenizers and stop- words, and a TfidfVectorizer to transform raw text data into a matrix of TF-IDF features [69]. The vectorizer was configured to remove common English stop words, exclude numbers, and include only alphabetic words. To limit the feature space, we set the min_df parameter to 3, excluding words that appeared in fewer than three documents, and capped the maximum number of features at 1000.

**Transformer models** We fine-tuned a series of pre-trained transformer-based models locally (DistilBERT) [70], optimizing hyperparameters with optuna [71]. Given the pre- processed text data, these models were used to predict 1. the true source of the explanation text, 2. the participant’s predicted source, and 3. the participant’s agreement with the judgment. Label classes were encoded for these multi-class and binary classification models.

### Semantic analysis

To understand which specific semantic features in the text could explain outcomes, we built a random forest classifier with 100 estimators trained on the corpora’s dense representation [72]. The decision to switch model architectures was made after confirming that performance scores were comparable across the transformer-based and tree-based implementations; the random forest implementation was less computationally intensive and easier to interpret through feature importance scores. To interpret the model’s predictions, we applied SHAP (SHapley Additive exPlanations) values using TreeExplainer [73]. SHAP values decompose predictions into contributions from individual features, providing insights into how different features influenced the model’s decisions. Positive SHAP values indicate that a feature contributes to a higher prediction, while negative values suggest a lower prediction.

## References

[1] Haupt CE, Marks M. AI-generated medical advice-GPT and beyond. JAMA. 2023;329(16):1349–50. doi: 10.1001/jama.2023.5321 36972070

[2] Cheong I, Xia K, Feng KJK, Chen QZ, Zhang AX. I Am Not a Lawyer, But…: Engaging Legal Experts Towards Responsible LLM Policies for Legal Advice. In: The 2024 ACM Conference on Fairness, Accountability, and Transparency, Rio de Janeiro, Brazil, 2024. p. 2454–69. 10.1145/3630106.3659048

[3] Marrapese A, Suleiman B, Ullah I, Kim J. A novel nuanced conversation evaluation framework for large language models in mental health. arXiv. 2024. http://arxiv.org/abs/2403.09705

[4] Chatterji A, Cunningham T, Deming DJ, Hitzig Z, Ong C, Shan CY. How people use chatgpt. 2025. [cited 2025 Oct 14]. https://www.nber.org/papers/w34255

[5] Freisinger E, Schneider S. Decoding decision delegation to artificial intelligence: a mixed-methods study on the preferences of decision-makers and decision-affected in surrogate decision contexts. European Management Journal. 2025;43(6):958–69. doi: 10.1016/j.emj.2024.10.004

[6] Doshi J, Novacic I, Fletcher C, Borges M, Zhong E, Marino MC. Sleeper social bots: a new generation of AI disinformation bots are already a political threat. arXiv. 2024. doi: 10.48550/arXiv.2408.12603

[7] Sun Y, He J, Cui L, Lei S, Lu CT. Exploring the deceptive power of LLM-generated fake news: a study of real-world detection challenges. arXiv. 2024. [cited 2024 Sept 12]. http://arxiv.org/abs/2403.18249

[8] Jones CR, Bergen BK. Does GPT-4 pass the Turing test?. arXiv. 2024. [cited 2024 June 27]. http://arxiv.org/abs/2310.20216

[9] Chiang WL, Zheng L, Sheng Y, Angelopoulos AN, Li T, Li D. Chatbot arena: an open platform for evaluating LLMs by human preference. arXiv. 2024. doi: 10.48550/arXiv.2403.04132

[10] Clark E, August T, Serrano S, Haduong N, Gururangan S, Smith NA. All that’s « human » is not gold: evaluating human evaluation of generated text. arXiv. 2021. doi: 10.48550/arXiv.2107.00061

[11] Köbis N, Mossink LD. Artificial intelligence versus Maya Angelou: experimental evidence that people cannot differentiate AI-generated from human-written poetry. Computers in Human Behavior. 2021;114:106553. doi: 10.1016/j.chb.2020.106553

[12] Porter B, Machery E. AI-generated poetry is indistinguishable from human-written poetry and is rated more favorably. Sci Rep. 2024;14(1):26133. doi: 10.1038/s41598-024-76900-1

[13] Kreps SE, McCain M, Brundage M. All the news that’s fit to fabricate: AI-generated text as a tool of media misinformation. In: SSRN Scholarly Paper, 2020. 10.2139/ssrn.3525002

[14] Jakesch M, Hancock JT, Naaman M. Human heuristics for AI-generated language are flawed. Proc Natl Acad Sci U S A. 2023;120(11):e2208839120. doi: 10.1073/pnas.2208839120 36881628 PMC10089155

[15] Awad E, Dsouza S, Kim R, Schulz J, Henrich J, Shariff A, et al. The moral machine experiment. Nature. 2018;563(7729):59–64. doi: 10.1038/s41586-018-0637-6 30356211

[16] Burton JW, Stein M, Jensen TB. A systematic review of algorithm aversion in augmented decision making. Behavioral Decision Making. 2019;33(2):220–39. doi: 10.1002/bdm.2155

[17] Castelo N, Bos MW, Lehmann DR. Task-dependent algorithm aversion. Journal of Marketing Research. 2019;56(5):809–25. doi: 10.1177/0022243719851788

[18] Cadario R, Longoni C, Morewedge CK. Understanding, explaining, and utilizing medical artificial intelligence. Nat Hum Behav. 2021;5(12):1636–42. doi: 10.1038/s41562-021-01146-0 34183800

[19] Logg JM, Minson JA, Moore DA. Algorithm appreciation: People prefer algorithmic to human judgment. Organizational Behavior and Human Decision Processes. 2019;151:90–103. doi: 10.1016/j.obhdp.2018.12.005

[20] De Freitas J, Agarwal S, Schmitt B, Haslam N. Psychological factors underlying attitudes toward AI tools. Nat Hum Behav. 2023;7(11):1845–54. doi: 10.1038/s41562-023-01734-2 37985913

[21] Kapania S, Siy O, Clapper G, Sp AM, Sambasivan N. ”Because AI is 100% right and safe”: user Attitudes and Sources of AI Authority in India. In: CHI Conference on Human Factors in Computing Systems. 2022. p. 1–18. 10.1145/3491102.3517533

[22] Dillion D, Mondal D, Tandon N, Gray K. AI language model rivals expert ethicist in perceived moral expertise. Sci Rep. 2025;15(1):4084. doi: 10.1038/s41598-025-86510-0 39900619 PMC11790977

[23] Howe PDL, Fay N, Saletta M, Hovy E. ChatGPT’s advice is perceived as better than that of professional advice columnists. Front Psychol. 2023;14:1281255. doi: 10.3389/fpsyg.2023.1281255 38078232 PMC10702579

[24] Ovsyannikova D, de Mello VO, Inzlicht M. Third-party evaluators perceive AI as more compassionate than expert humans. Commun Psychol. 2025;3(1):4. doi: 10.1038/s44271-024-00182-6 39794410 PMC11723910

[25] Proksch S, Schühle J, Streeb E, Weymann F, Luther T, Kimmerle J. The impact of text topic and assumed human vs. AI authorship on competence and quality assessment. Front Artif Intell. 2024;7:1412710. doi: 10.3389/frai.2024.1412710 38881953 PMC11176609

[26] Böhm R, Jörling M, Reiter L, Fuchs C. People devalue generative AI’s competence but not its advice in addressing societal and personal challenges. Commun Psychol. 2023;1(1):32. doi: 10.1038/s44271-023-00032-x 39242905 PMC11332189

[27] Zhang Y, Gosline R. Human favoritism, not AI aversion: People’s perceptions (and bias) toward generative AI, human experts, and human–GAI collaboration in persuasive content generation. Judgm decis mak. 2023;18. doi: 10.1017/jdm.2023.37

[28] Morewedge CK. Preference for human, not algorithm aversion. Trends Cogn Sci. 2022;26(10):824–6. doi: 10.1016/j.tics.2022.07.007 35941064

[29] Bigman YE, Gray K. People are averse to machines making moral decisions. Cognition. 2018;181:21–34. doi: 10.1016/j.cognition.2018.08.003 30107256

[30] Hendrycks D, Burns C, Basart S, Critch A, Li J, Song D. Aligning AI with shared human values. arXiv. 2021. doi: 10.48550/arXiv.2008.02275

[31] Kazim E, Koshiyama AS. A high-level overview of AI ethics. Patterns (N Y). 2021;2(9):100314. doi: 10.1016/j.patter.2021.100314 34553166 PMC8441585

[32] Scherrer N, Shi C, Feder A, Blei DM. Evaluating the moral beliefs encoded in LLMs. arXiv. 2023. [cited 2024 June 26]. http://arxiv.org/abs/2307.14324

[33] Sachdeva PS, van Nuenen T. Normative evaluation of large language models with everyday moral dilemmas. arXiv.org. 2025. [cited 2025 Feb 6]. https://arxiv.org/abs/2501.18081v1

[34] Park PS, Schoenegger P, Zhu C. Correct answers from the psychology of artificial intelligence. arXiv. 2023. doi: 10.48550/arXiv.2302.07267

[35] Zhu JQ, Yan H, Griffiths TL. Language models trained to do arithmetic predict human risky and intertemporal choice. arXiv. 2024. doi: 10.48550/arXiv.2405.19313

[36] Aharoni E, Fernandes S, Brady DJ, Alexander C, Criner M, Queen K, et al. Attributions toward artificial agents in a modified Moral Turing Test. Sci Rep. 2024;14(1):8458. doi: 10.1038/s41598-024-58087-7 38688951 PMC11061136

[37] Osborne MR, Bailey ER. Me vs. the machine? Subjective evaluations of human- and AI-generated advice. Sci Rep. 2025;15(1):3980. doi: 10.1038/s41598-025-86623-6 39893236 PMC11787321

[38] Babin JJ, Chauhan H. Chatbot or Humanaut? How the Source of Advice Impacts Behavior in One-shot Social Dilemmas. 2024. [cited 2025 Jan 23]. https://www.researchsquare.com/article/rs-4462506/latest

[39] Mitrović S, Andreoletti D, Ayoub O. ChatGPT or Human? Detect and Explain. Explaining Decisions of Machine Learning Model for Detecting Short ChatGPT-generated Text. arXiv. 2023. doi: 10.48550/arXiv.2301.13852

[40] Koenigs M, Young L, Adolphs R, Tranel D, Cushman F, Hauser M, et al. Damage to the prefrontal cortex increases utilitarian moral judgements. Nature. 2007;446(7138):908–11. doi: 10.1038/nature05631 17377536 PMC2244801

[41] Greene JD, Nystrom LE, Engell AD, Darley JM, Cohen JD. The neural bases of cognitive conflict and control in moral judgment. Neuron. 2004;44(2):389–400. doi: 10.1016/j.neuron.2004.09.027 15473975

[42] Brown TB, Mann B, Ryder N, Subbiah M, Kaplan J, Dhariwal P, et al. Language Models are Few-Shot Learners. arXiv. 2020. doi: 10.48550/arXiv.2005.14165

[43] Achiam J, Adler S, Agarwal S, Ahmad L, Akkaya I. OpenAI. GPT-4 Technical Report. 2024. doi: 10.48550/arXiv.2303.08774

[44] Cushman F, Young L, Hauser M. The role of conscious reasoning and intuition in moral judgment: testing three principles of harm. Psychol Sci. 2006;17(12):1082–9. doi: 10.1111/j.1467-9280.2006.01834.x 17201791

[45] Hagendorff T, Dasgupta I, Binz M, Chan SCY, Lampinen A, Wang JX. Machine psychology. arXiv. 2024. [cited 2024 Aug 25]. http://arxiv.org/abs/2303.13988

[46] Yax N, Anlló H, Palminteri S. Studying and improving reasoning in humans and machines. Commun Psychol. 2024;2(1):51. doi: 10.1038/s44271-024-00091-8 39242743 PMC11332180

[47] Carmona-Perera M, Vilar-Lopez R, Perez-Garcia M, Verdejo-Garcia A. Using moral dilemmas to characterize social decision-making. Clinical Neuropsychiatry. 2013;10(2).

[48] Holyoak KJ, Powell D. Deontological coherence: a framework for commonsense moral reasoning. Psychol Bull. 2016;142(11):1179–203. doi: 10.1037/bul0000075 27709981

[49] Moore AB, Lee NYL, Clark BAM, Conway ARA. In defense of the personal/impersonal distinction in moral psychology research: cross-cultural validation of the dual process model of moral judgment. Judgm decis mak. 2011;6(3):186–95. doi: 10.1017/s193029750000139x

[50] Myers S, Everett JAC. People expect artificial moral advisors to be more utilitarian and distrust utilitarian moral advisors. Cognition. 2025;256:106028. doi: 10.1016/j.cognition.2024.106028 39671980

[51] Binz M, Schulz E. Using cognitive psychology to understand GPT-3. Proc Natl Acad Sci U S A. 2023;120(6):e2218523120. doi: 10.1073/pnas.2218523120 36730192 PMC9963545

[52] Hagendorff T, Fabi S, Kosinski M. Human-like intuitive behavior and reasoning biases emerged in large language models but disappeared in ChatGPT. Nat Comput Sci. 2023;3(10):833–8. doi: 10.1038/s43588-023-00527-x 38177754 PMC10766525

[53] Khamassi M, Nahon M, Chatila R. Strong and weak alignment of large language models with human values. Sci Rep. 2024;14(1):19399. doi: 10.1038/s41598-024-70031-3 39169090 PMC11339283

[54] Kahane G. On the wrong track: process and content in moral psychology. Mind Lang. 2012;27(5):519–45. doi: 10.1111/mila.12001 23335831 PMC3546390

[55] Mariadassou S, Klesse A-K, Boegershausen J. Averse to what: consumer aversion to algorithmic labels, but not their outputs?. Curr Opin Psychol. 2024;58:101839. doi: 10.1016/j.copsyc.2024.101839 38996629

[56] Ishowo-Oloko F, Bonnefon JF, Soroye Z, Crandall J, Rahwan I, Rahwan T. Behavioural evidence for a transparency–efficiency tradeoff in human–machine cooperation. Nat Mach Intell. 2019;1(11):517–21. doi: 10.1038/s42256-019-0113-5

[57] Rahman MJ, Liang H, Xue Y. AI aversion: a task dependent multigroup analysis. In: PACIS 2023 Proceedings, 2023. https://aisel.aisnet.org/pacis2023/86

[58] Allen C, Varner G, Zinser J. Prolegomena to any future artificial moral agent. Journal of Experimental & Theoretical Artificial Intelligence. 2000;12(3):251–61. doi: 10.1080/09528130050111428

[59] Hewstone M, Rubin M, Willis H. Intergroup bias. Annual Review of Psychology. 2002;53(1):575–604. doi: 10.1146/annurev.psych.53.100901.135109 11752497

[60] Baker LJ, Li H, Hammond H, Jaeger CB, Havard A, Lane JD, et al. The roles of cognitive dissonance and normative reasoning in attributions of minds to robots. Cogn Res Princ Implic. 2024;9(1):80. doi: 10.1186/s41235-024-00604-3 39663309 PMC11635073

[61] Bauer K, von Zahn M, Hinz O. Expl(AI)ned: The impact of explainable artificial intelligence on cognitive processes. 315. 2022. https://ideas.repec.org//p/zbw/safewp/315.html

[62] Festinger L. Cognitive dissonance. Scientific American. 1962;207(4):93–106. 13892642 10.1038/scientificamerican1062-93

[63] Denia E. AI narratives model: Social perception of artificial intelligence. Technovation. 2025;146:103266. doi: 10.1016/j.technovation.2025.103266

[64] Bilancini E, Boncinelli L, Capraro V, Celadin T, Paolo RD. “Do the right thing” for whom? An experiment on ingroup favouritism, group assorting and moral suasion. Judgm decis mak. 2020;15(2):182–92. doi: 10.1017/s1930297500007336

[65] Leidner B, Castano E, Zaiser E, Giner-Sorolla R. Ingroup glorification, moral disengagement, and justice in the context of collective violence. Pers Soc Psychol Bull. 2010;36(8):1115–29. doi: 10.1177/0146167210376391 20693388

[66] Welivita A, Pu P. Are Large Language Models More Empathetic than Humans? [Internet]. arXiv; 2024 [cited 3 Sept 2024]. doi: 10.48550/arXiv.2406.05063

[67] Hui BP, Lau CL, Sun R, R LC, Hendra LB, Kogan A. Decoding moral reasoning in AI: a quantitative analysis of few-shot learning models of GPT. In: 2024. https://papers.ssrn.com/sol3/papers.cfm?abstract_id=5019221

[68] Keshmirian A, Baltaji R, Hemmatian B, Asghari H, Varshney LR. Many LLMs are more utilitarian than one. arXiv. 2025. doi: 10.48550/arXiv.2507.00814

[69] Qaiser S, Ali R. Text mining: use of TF-IDF to examine the relevance of words to documents. IJCA. 2018;181(1):25–9. doi: 10.5120/ijca2018917395

[70] Sanh V, Debut L, Chaumond J, Wolf T. DistilBERT, a distilled version of BERT: smaller, faster, cheaper and lighter. arXiv. 2020. doi: 10.48550/arXiv.1910.01108

[71] Akiba T, Sano S, Yanase T, Ohta T, Koyama M. Optuna. Proceedings of the 25th ACM SIGKDD International Conference on Knowledge Discovery & Data Mining. 2019;2623–31. doi: 10.1145/3292500.3330701

[72] Breiman L. Random forests. Machine learning. 2001;45(1):5–32. doi: 10.1023/A:1010933404324

[73] Lundberg SM, Lee SI. A unified approach to interpreting model predictions. In: Proceedings of the 31st International Conference on Neural Information Processing Systems. 2017. p. 4768–77.

