# Supplementary material for: A moral Turing test: How belief and source shape detection of and agreement with LLM judgments
Source: PLoS One. 2026 Aug 5;21(8):e0353391. doi: 10.1371/journal.pone.0353391 (PMC13440878; doi:10.1371/journal.pone.0353391)
Supplement: Supplementary file 1 [file pone.0353391.s002.docx]

### Dear Editor and Reviewers,

### We would like to thank you for your time, careful review, and constructive feedback on our manuscript. We greatly appreciate your insightful comments, which have helped us clarify our methodology, reorganize our structure for better readability, and strengthen our overall discussion. Below, we provide a point-by-point response (in blue) to each of the suggestions provided by Reviewer #1 and Reviewer #2, detailing the specific revisions we have made to the manuscript.

### **Reviewer #1**

**1.** "Consider identifying the research vocabulary clearly and in a sequential integrated manner, an (e.g. an Introduction - line 36 - cannot be followed by Results – line 123) even if you wish to keep your own titles."

"Reading your work leads to grasp three procedures (or phases, or experiments): primary questionnaire, secondary questionnaire, and a final language analysis; with each of the three having their own analysis and findings. Whether this is correct or not- the main body needs to be clearly recognized in terms of research vocabulary."

We added an “overview of the study” subsection to smoothen the transition from introduction to results.

“

**Overview of the study**

To systematically investigate human perceptions of AI moral decision-making, our study was conducted in three main phases. First, in the corpus generation phase, we collected judgments and textual justifications for a diverse set of moral and non-moral scenarios from both human participants and large language models (specifically, GPT-3.5 variants). Second, in the corpus evaluation phase, we presented these responses to new, independent groups of human evaluators who were tasked with identifying the authorship source (AI or human) and indicating their level of agreement with both the judgment and the justification. Finally, we performed extensive linguistic and semantic analyses, utilizing NLP techniques, predictive modeling, and SHAP interpretations, to isolate the specific textual features (such as formal reasoning cues and cost-benefit markers) that drove participants' detection accuracy and moral alignment. Full methodological details are provided in the Methods section.

“

2. "When first mentioned in the text, consider clarifying the main sources of the (footbridge- trolley) scenarios – also known as the fat man scenario- and adding two lines to present what they are- even if they are publicly known, may be also referring to your Fig 2A - if possible."

We added this short description to the Introduction section (line 104-107):

"For instance, in the classic 'trolley problem,' participants decide whether to divert a runaway trolley to save five people at the cost of one (an impersonal dilemma). In the 'footbridge' variant, participants must decide whether to physically push a person off a bridge to stop the trolley (a personal dilemma; see Figure 2A for examples across scenario types)."

3. "Table 1 seems to be a master table. It is referred to starting on page 12 but only appears on page 20, while constantly referring to its part between the two pages. To keep a more fluent readability, it may be more effective to present it earlier. An alternative solution is to break it into smaller tables following each related section. Perhaps, to recheck other figures and tables."

We have broken the master Table 1 into smaller, section-specific tables (Table 1, Table 2, Table 3, etc.) and embedded them directly after the corresponding paragraphs. We thank the reviewer for this suggestion as it greatly improved the readability.

4. "Consider categorizing the (Discussion) section into more than one section, following the findings type, or any manner you find it sound and suitable for your work. It helps tracking the sequence of the whole work."

In the discussion, we added thematic subheadings to organize the flow of the findings: “Scenario complexity modulates preference for AI judgments, Belief-driven anti-AI bias overrides actual source, Preferred but unattributed: the LLM authorship paradox, In-group/out-group bias as an explanatory mechanism, Surface linguistic features drive detection alone, Semantic reasoning markers shape both detection and agreement, Limitations and moral-alignment caveats, Residual detectability despite humanization, Dissociating moral agency from moral judgment quality, LLMs as persuasive but unacknowledged moral advisors. “

5. "The (Method) section - line 569 – normally comes at the first part of the text - before the main text body, to help the reader grasping the (big picture) of the research, and rationalize the overall structure of the experiment... Perhaps you would consider a transition between the introductions of the (steps – line 570) mentioned in this section – lines from 572-592, and their full explanation sections – starting at line 596. Perhaps also consider numbering these steps or use bullets to identify them."

We follow a structure common in PLOS ONE publications, where detailed Methods appear after Results. To improve readability, we have added a Study Overview paragraph between Introduction and Results sections.

We have numbered the six steps in the Methods overview (Step 1: Corpus generation, Step 2: Corpus transformation, and so on) and added brief transition sentences between them to improve the flow and readability of this section. These changes can be found in the revised Methods section.

6. "Language adjustments: o Perhaps you may consider shortening the length of the main title. o It will help to review for typing errors (e.g. lines 90, 91 – to check the parentheses consistency). o Consider the referencing style method (e.g. references 41 and 42 has a long list of authors, the style would shorten list). o Consider not repeating titles and subtitles (e.g. Corpus generation – lines 124, and 572/596). o May be to consider shortening the long subtitles (e.g. subtitles: “Detection is above chance and enhanced in morally engaging scenarios” – could be changed, for example to “Detection effects on moral scenarios”;"

We thank the reviewer for their careful reading and constructive suggestions. We have addressed each point as follows.

We have revised the title. The new title now reads: *"A Moral Turing Test: How Belief and Source Shape Detection of and Agreement with LLM Judgments."* We believe this retains the key information while being more concise.

Regarding typos**,** we have proofread the entire manuscript. The parentheses inconsistency at lines 90–91 has been corrected, along with other minor typographical errors identified during this review.

References 41 and 42 have been reformatted to list the first six authors followed by "et al.," in accordance with PLOS ONE reference style guidelines.

We have renamed the relevant subsections to eliminate ambiguity. The Results subsection is now titled "LLMs replicate the moral framing effect " instead of corpus generation.

We have shortened the subtitles as suggested. For example, "Detection is above chance and enhanced in morally engaging scenarios" is now "Detection accuracy across scenario types"; "Agreement is shaped by both the scenario type and the belief about the source" is now "Agreement as a function of source and belief"; and so on. We believe these revised subtitles are more concise while preserving their informational content.

Reviewer #2

1. "Introduction: The discussion of the literature shows that the area is complex and nuanced, with a variety of views and results. (On line 434 you refer to "the standard theory": what is this, and is there one really?)"

We agree that "the standard theory" overclaimed consensus. We have replaced "the standard theory predicts that" with "dual-process accounts of moral cognition predict that" in the Discussion, attributing the framework explicitly to Greene and colleagues. The existing caveat to Kahane (ref. 53) in the following sentence already acknowledges that this framework is debated, and we have kept that caveat in place.

2. "It would be good if the paper took a clearer stance on certain key issues, since assumptions seem to be made about them throughout but are not always explicit. In particular, when and why is it crucial for people to be able to identify the source of something as AI? When does confusion about the source present a particular risk?... When might it be appropriate to prefer the judgement of an AI system?"

We have added a new paragraph in the Introduction laying out our working position:

“Taken together, these findings suggest the human perception of machine judgment can go in two opposite directions. A preference for AI may occur when machines are seen as authoritative sources of knowledge. In contrast, an aversion for AI might emerge from societal or psychological prejudices against machines, often stemming from the belief that machines lack agency and the capacity for compassionate or morally sound decisions (*29*). Ultimately, the importance of source identification depends heavily on the context of the interaction. Knowing whether content is AI- or human-generated becomes highly consequential when accountability is at stake, when the content is advisory rather than purely informational, and when users might calibrate their trust differently based on the author. While deferring to AI may be less problematic for pattern-recognition tasks with a clear ground truth, it becomes far more complex when the grounds for judgment are themselves contested. Moral preferences are precisely such a case: they capture ethically consequential scenarios (*30*, *31*) where the basis for judgment is inherently subjective, making them a critical testing ground for examining the detection and acceptance of machine judgments by humans.”

3. "The discussion of the literature could usefully indicate that much is uncertain or provisional... LLMs' justifications of their judgements are likely to be very much better since GPT5.x (especially where x >1) compared with GPT3.5. Any study in this area (including the present paper) can provide only a snapshot." "514: in particular, might not generalise to more recent versions of GPT."

We have strengthened the Limitations paragraph to make the snapshot nature of the findings explicit. The paragraph now states that "the quality of LLM-generated moral justifications has increased substantially in more recent model families (e.g., GPT-4, GPT-5 and their successors). Our results should therefore be read as a snapshot tied to this generation of models. In particular, detection accuracy, the magnitude of the pro-AI bias in personal scenarios, and the persistence of the belief-driven anti-AI bias are all empirical questions that should be re-examined with newer model families." This flags both the generational limitation and the specific quantities that we expect to shift.

4. "It might be useful to have a sub-heading at line 98, where the study itself starts to be described."

We have added a "The present study" sub-heading immediately before the paragraph beginning "To investigate human perceptions [...]".

5. "Line 85: "linguistic features play a significant role" — "Thus" before this seems misplaced because the previous text isn't clearly about linguistic features. Really it needs the context of the discussion later in the paper."

We have deleted "Thus," and replaced it with "These findings suggest that ", which better reflects the actual logical relation between the preceding discussion of model-level alignment and the forward-pointing claim about linguistic features that we analyze later in the paper.

6. "99: Who were the participants? Social profile, age, gender etc.? These aspects could bear on their pattern of responses, e.g. relative to other studies. [I see this occurs in the method section: a cross-reference here would be useful.]"

We have added a cross-reference parenthetical immediately after the mention of 230 participants in the Introduction: "Full demographic details (age, gender, country) are reported in the Methods (see *Participants*)." This lets readers locate the demographic information without duplicating it in the Introduction.

7. "103: It's confusing here (and sometimes elsewhere) to conflate deontological with emotional. Deontological responses would come from specific, often absolute moral principles; emotion seems on the whole more likely to relate to somewhat arbitrary and case-specific responses. Not everything that is non-consequentialist is deontological." "407-8: again, it is wrong to conflate the personal and the deontological, if that's what's suggested here."

We have added a clarifying sentence right after the "deontological-like consideration" phrase in the Introduction: "We note that refusals in personal moral scenarios may reflect emotionally-driven aversion rather than principle-based (i.e., strictly deontological) reasoning; throughout the paper we reserve the term 'deontological' for principle-based judgments and use 'non-utilitarian' where the distinction matters." This explicitly separates the two constructs and signals the terminological convention we follow in the rest of the paper.

8. "156-9: The "folk-psychology intuition" seems to be ungrounded for LLMs, prior to considering your results. One might expect that LLMs would mirror the dominant patterns in their training material..."

We have rewritten the sentence to reframe the finding as an empirical test of the lay intuition rather than a direct challenge to a supposedly dominant theoretical claim. The sentence now reads: "Overall, our results empirically test, and do not support, the popular lay intuition that LLMs reason in a more utilitarian fashion than humans: in our data the LLMs we tested were no more utilitarian than human participants, and davinci-003 was in fact less so in personal moral scenarios." This positions the utilitarian-AI expectation as a folk belief to be tested, which is what it was.

9. "175-6: it would be good to know more about exactly how this incentivisation worked."

We have added a parenthetical right at the first mention of incentivization in the Results: "(a bonus of 5¢ per correct identification, yielding an average bonus of £1.46; see *Participants* in the Methods)". Readers now get the key quantitative detail inline without having to hunt for it in the Methods.

10. "200-206: This point about agreement with the judgement vs agreement with the justification is important, but somehow often underplayed. It's not always clear when "agreement" means with the one or the other, or perhaps both."

We have surfaced the operational definition of agreement early in the Results, rather than leaving it buried in the Methods. The inserted sentence reads: "Note that throughout the paper 'agreement' refers to the mean of two binary items collected for each response: agreement with the judgment (yes/no decision) and agreement with the justification (free-text reasoning), coded 1/0.5/0 for full agreement, partial agreement (agreement with one but not the other), and full disagreement (see Methods, *Statistical evaluation*). The two components move together in our data, but we flag divergences where they occur." This removes the ambiguity about which quantity "agreement" refers to.

11. "244-5: potential ambiguity of "significant" — the p-value is significant, but is the detection rate above 50% to a significant extent? (Cf. point about line 487 below.)"

We have rewritten the sentence to distinguish statistical significance from effect magnitude. The sentence now reads: "Overall, detection was statistically above chance but modest in absolute terms. In dv2 corpus (humans and davinci-002 justifications), participants correctly identified the source 64% of the time..." The word "significant" is no longer doing double duty, and the same clarification has been applied at the corresponding point later in the Discussion.

12. "252-3: this point about "acceptability" seems to require a little more clarification as to whether the acceptability of the judgement is due to the linguistic quality (presumably of the justification) driving ideas about its source, or is determined irrespective of this."

We have added a sentence making the causal structure explicit: "In our data, low-level linguistic features (length, typos, first-person pronouns) predicted participants' beliefs about the source but were not themselves associated with agreement; acceptability is thus better described as being mediated through source inference than as being a direct response to linguistic quality." This states which of the two mediation paths our data actually support.

13. "449-50: but the findings you've just mentioned do seem to suggest a simple aversion to AI (or pro-human bias, which appears to be the same thing)? (Also, in this sentence, there are two groups of citation numbers: what do they each relate to? Why are there two different groups?)"

We thank the reviewer for pointing this out. First, regarding the formatting, we agree and have grouped the citations together in the revised manuscript, as it indeed makes more sense and improves readability.

Second, regarding the distinction between AI aversion and pro-human bias: in the context of our specific data, we remain agnostic as to whether our observed effect is caused strictly by the former or the latter; it is entirely possible that it is a mix of both. However, we maintained the distinction in the text because the recent literature increasingly treats these as separable mechanisms rather than mere synonyms (e.g., Osborne & Bailey, 2025; Zhang & Gosline, 2023). As we elaborate later in the Discussion, these conceptually represent distinct psychological mechanisms that produce similar behavioral outcomes. AI aversion is defined by a source-based distrust or rejection of algorithms, even when they perform well. In contrast, pro-human bias stems from a positive preference for human authenticity and in-group identity rather than a generalized dislike of machines.

14. "508-9: Do you mean a cue to the judgement being utilitarian or it being AI-generated? These are not the same thing, even if people's biases about them may tend to align."

We have rewritten the passage to separate the two cues explicitly: "It is important to note that cost-benefit vocabulary functions as two distinct cues: (i) a marker of utilitarian reasoning within the content itself, and (ii), independently, a folk-psychological cue to AI authorship. Empirically in our data these came apart: the LLMs we tested were not more utilitarian than humans, yet participants still used this vocabulary to infer AI authorship." This spells out the decoupling, which, as the reviewer notes, is one of the more interesting findings of the paper.

15. "535-541: why is there a tension or dissonance here? Or, what kind of tension is it? Suppose we agree that AI agents have no moral agency, this seems to be entirely"

We have softened "tension" and "dissonance" throughout this passage, replacing them with "dissociation" and "asymmetry" (the latter being consistent with the existing subsection heading "Dissociating moral agency from moral judgment quality"). The passage now characterizes the phenomenon as an observed asymmetry between attributed moral agency and perceived judgment quality, without presupposing the stronger psychological claim of dissonance that the reviewer rightly noted is not established by our data.

16. "621: what are ambiguous answers and why were they removed? (Is ambiguity in moral judgement a bad thing? If so, why?)"

We have added an operational definition to the Methods: "[ambiguous answers,] defined as responses where the binary yes/no judgment could not be determined from the free-text justification (e.g., 'maybe,' 'it depends,' or replies that argued both sides without committing to a judgment); we are not here removing morally ambiguous scenarios but uncodable responses". This clarifies that the exclusion was coding-driven, not philosophical; moral ambiguity in the scenarios themselves is preserved, and only responses whose binary judgment could not be extracted were removed.
