# Supplementary material for: A moral Turing test: How belief and source shape detection of and agreement with LLM judgments
Source: PLoS One. 2026 Aug 5;21(8):e0353391. doi: 10.1371/journal.pone.0353391 (PMC13440878; doi:10.1371/journal.pone.0353391)
Supplement: Peer review history

# Peer review: Decision Letter 0

**DOI:** 10.1371/journal.pone.0353391.r001

**Authors:** Stephen R. Milford

3 Apr 2026

PONE-D-26-10105

A Moral Turing Test to Assess How Subjective Belief and Objective Source Affect Detection and Agreement with LLM Judgments

PLOS One

Dear Dr. Garcia,

Thank you for submitting your manuscript to PLOS ONE. After careful consideration, we feel that it has merit but does not fully meet PLOS ONE’s publication criteria as it currently stands. Therefore, we invite you to submit a revised version of the manuscript that addresses the points raised during the review process.

Please submit your revised manuscript by May 18 2026 11:59PM. If you will need more time than this to complete your revisions, please reply to this message or contact the journal office at plosone@plos.org. When you're ready to submit your revision, log on to https://www.editorialmanager.com/pone/ and select the 'Submissions Needing Revision' folder to locate your manuscript file.

Please include the following items when submitting your revised manuscript:

- A letter that responds to each point raised by the academic editor and reviewer(s). You should upload this letter as a separate file labeled 'Response to Reviewers'.
- A marked-up copy of your manuscript that highlights changes made to the original version. You should upload this as a separate file labeled 'Revised Manuscript with Track Changes'.
- An unmarked version of your revised paper without tracked changes. You should upload this as a separate file labeled 'Manuscript'.

If you would like to make changes to your financial disclosure, please include your updated statement in your cover letter. Guidelines for resubmitting your figure files are available below the reviewer comments at the end of this letter.

If applicable, we recommend that you deposit your laboratory protocols in protocols.io to enhance the reproducibility of your results. Protocols.io assigns your protocol its own identifier (DOI) so that it can be cited independently in the future. For instructions see: https://journals.plos.org/plosone/s/submission-guidelines#loc-laboratory-protocols. Additionally, PLOS ONE offers an option for publishing peer-reviewed Lab Protocol articles, which describe protocols hosted on protocols.io. Read more information on sharing protocols at https://plos.org/protocols?utm_medium=editorial-email&utm_source=authorletters&utm_campaign=protocols.

As the corresponding author, your ORCID iD is verified in the submission system and will appear in the published article. PLOS supports the use of ORCID, and we encourage all coauthors to register for an ORCID iD and use it as well. Please encourage your coauthors to verify their ORCID iD within the submission system before final acceptance, as unverified ORCID iDs will not appear in the published article. *Only*  the individual author can complete the verification step; PLOS staff *cannot*  verify ORCID iDs on behalf of authors.

We look forward to receiving your revised manuscript.

Kind regards,

Stephen R. Milford

Academic Editor

PLOS One

**Journal Requirements:**

When submitting your revision, we need you to address these additional requirements.

1. Please ensure that your manuscript meets PLOS ONE's style requirements, including those for file naming. The PLOS ONE style templates can be found at

https://journals.plos.org/plosone/s/file?id=wjVg/PLOSOne_formatting_sample_main_body.pdf and

https://journals.plos.org/plosone/s/file?id=ba62/PLOSOne_formatting_sample_title_authors_affiliations.pdf

2. Please note that PLOS One has specific guidelines on code sharing for submissions in which author-generated code underpins the findings in the manuscript. In these cases, we expect all author-generated code to be made available without restrictions upon publication of the work. Please review our guidelines at https://journals.plos.org/plosone/s/materials-and-software-sharing#loc-sharing-code and ensure that your code is shared in a way that follows best practice and facilitates reproducibility and reuse.

3. We note that the grant information you provided in the ‘Funding Information’ and ‘Financial Disclosure’ sections do not match.

When you resubmit, please ensure that you provide the correct grant numbers for the awards you received for your study in the ‘Funding Information’ section.

4. Thank you for stating the following financial disclosure:

“S.P. is funded by the European Research Council consolidator grant (RaReMem: 101043804) and three Agence Nationale de la Recherche grants (CogFinAgent: ANR-21-CE23- 0002-02; RELATIVE: ANR-21-CE37- 750 0008-01; RANGE: ANR-21-CE28-0024-01), the Alexander Von Humbolt foundation and a Google unrestricted gift.”

Please state what role the funders took in the study.  If the funders had no role, please state: "The funders had no role in study design, data collection and analysis, decision to publish, or preparation of the manuscript."

If this statement is not correct you must amend it as needed.

Please include this amended Role of Funder statement in your cover letter; we will change the online submission form on your behalf.

5. Please note that funding information should not appear in any section or other areas of your manuscript. We will only publish funding information present in the Funding Statement section of the online submission form. Please remove any funding-related text from the manuscript.

6. Please note that your Data Availability Statement is currently missing the repository name. If your manuscript is accepted for publication, you will be asked to provide these details on a very short timeline. We therefore suggest that you provide this information now, though we will not hold up the peer review process if you are unable.

7. Thank you for stating the following in the Financial Disclosure section:

“S.P. is funded by the European Research Council consolidator grant (RaReMem: 101043804) and three Agence Nationale de la Recherche grants (CogFinAgent: ANR-21-CE23- 0002-02; RELATIVE: ANR-21-CE37- 750 0008-01; RANGE: ANR-21-CE28-0024-01), the Alexander Von Humbolt foundation and a Google unrestricted gift.”

We note that one or more of the authors are employed by a commercial company: Google LLC.

1. Please provide an amended Funding Statement declaring this commercial affiliation, as well as a statement regarding the Role of Funders in your study. If the funding organization did not play a role in the study design, data collection and analysis, decision to publish, or preparation of the manuscript and only provided financial support in the form of authors' salaries and/or research materials, please review your statements relating to the author contributions, and ensure you have specifically and accurately indicated the role(s) that these authors had in your study. You can update author roles in the Author Contributions section of the online submission form.

Please also include the following statement within your amended Funding Statement.

“The funder provided support in the form of salaries for authors [insert relevant initials], but did not have any additional role in the study design, data collection and analysis, decision to publish, or preparation of the manuscript. The specific roles of these authors are articulated in the ‘author contributions’ section.”

If your commercial affiliation did play a role in your study, please state and explain this role within your updated Funding Statement.

2. Please also provide an updated Competing Interests Statement declaring this commercial affiliation along with any other relevant declarations relating to employment, consultancy, patents, products in development, or marketed products, etc.

Within your Competing Interests Statement, please confirm that this commercial affiliation does not alter your adherence to all PLOS ONE policies on sharing data and materials by including the following statement: "This does not alter our adherence to  PLOS ONE policies on sharing data and materials.” (as detailed online in our guide for authors http://journals.plos.org/plosone/s/competing-interests) . If this adherence statement is not accurate and  there are restrictions on sharing of data and/or materials, please state these. Please note that we cannot proceed with consideration of your article until this information has been declared.

Please include both an updated Funding Statement and Competing Interests Statement in your cover letter. We will change the online submission form on your behalf.

8. We note that Figure 2c, 4, 7, and 8 in your submission contain copyrighted images. All PLOS content is published under the Creative Commons Attribution License (CC BY 4.0), which means that the manuscript, images, and Supporting Information files will be freely available online, and any third party is permitted to access, download, copy, distribute, and use these materials in any way, even commercially, with proper attribution. For more information, see our copyright guidelines: http://journals.plos.org/plosone/s/licenses-and-copyright.

We require you to either (1) present written permission from the copyright holder to publish these figures specifically under the CC BY 4.0 license, or (2) remove the figures from your submission:

1. You may seek permission from the original copyright holder of Figure 2c, 4, 7, and to publish the content specifically under the CC BY 4.0 license.

We recommend that you contact the original copyright holder with the Content Permission Form (http://journals.plos.org/plosone/s/file?id=7c09/content-permission-form.pdf) and the following text:

“I request permission for the open-access journal PLOS ONE to publish XXX under the Creative Commons Attribution License (CCAL) CC BY 4.0 (http://creativecommons.org/licenses/by/4.0/). Please be aware that this license allows unrestricted use and distribution, even commercially, by third parties. Please reply and provide explicit written permission to publish XXX under a CC BY license and complete the attached form.”

Please upload the completed Content Permission Form or other proof of granted permissions as an "Other" file with your submission.

In the figure caption of the copyrighted figure, please include the following text: “Reprinted from [ref] under a CC BY license, with permission from [name of publisher], original copyright [original copyright year].”

2. If you are unable to obtain permission from the original copyright holder to publish these figures under the CC BY 4.0 license or if the copyright holder’s requirements are incompatible with the CC BY 4.0 license, please either i) remove the figure or ii) supply a replacement figure that complies with the CC BY 4.0 license. Please check copyright information on all replacement figures and update the figure caption with source information. If applicable, please specify in the figure caption text when a figure is similar but not identical to the original image and is therefore for illustrative purposes only.

9. Please remove your figures from within your manuscript file, leaving only the individual TIFF/EPS image files, uploaded separately. These will be automatically included in the reviewers’ PDF.

10. If the reviewer comments include a recommendation to cite specific previously published works, please review and evaluate these publications to determine whether they are relevant and should be cited. There is no requirement to cite these works unless the editor has indicated otherwise.

11. Please review your reference list to ensure that it is complete and correct. If you have cited papers that have been retracted, please include the rationale for doing so in the manuscript text, or remove these references and replace them with relevant current references. Any changes to the reference list should be mentioned in the rebuttal letter that accompanies your revised manuscript. If you need to cite a retracted article, indicate the article’s retracted status in the References list and also include a citation and full reference for the retraction notice.

[Note: HTML markup is below. Please do not edit.]

Reviewers' comments:

Reviewer's Responses to Questions

**Comments to the Author**

1. Is the manuscript technically sound, and do the data support the conclusions?

The manuscript must describe a technically sound piece of scientific research with data that supports the conclusions. Experiments must have been conducted rigorously, with appropriate controls, replication, and sample sizes. The conclusions must be drawn appropriately based on the data presented.

Reviewer #1: Yes

Reviewer #2: Yes

**********

2. Has the statistical analysis been performed appropriately and rigorously?

Reviewer #1: Yes

Reviewer #2: I Don't Know

**********

3. Have the authors made all data underlying the findings in their manuscript fully available?

The PLOS Data policy requires authors to make all data underlying the findings described in their manuscript fully available without restriction, with rare exception (please refer to the Data Availability Statement in the manuscript PDF file). The data should be provided as part of the manuscript or its supporting information, or deposited to a public repository. For example, in addition to summary statistics, the data points behind means, medians and variance measures should be available. If there are restrictions on publicly sharing data—e.g. participant privacy or use of data from a third party—those must be specified.

Reviewer #1: Yes

Reviewer #2: Yes

**********

4. Is the manuscript presented in an intelligible fashion and written in standard English?

PLOS ONE does not copyedit accepted manuscripts, so the language in submitted articles must be clear, correct, and unambiguous. Any typographical or grammatical errors should be corrected at revision, so please note any specific errors here.

Reviewer #1: No

Reviewer #2: Yes

**********

5. Review Comments to the Author

Please use the space provided to explain your answers to the questions above. You may also include additional comments for the author, including concerns about dual publication, research ethics, or publication ethics. (Please upload your review as an attachment if it exceeds 20,000 characters)

Reviewer #1: This work could be strengthened with the following minor adjustments:

Organization adjustments:

• Consider identifying the research vocabulary clearly and in a sequential integrated manner, an (e.g. an Introduction - line 36 - cannot be followed by Results – line 123) even if you wish to keep your own titles.

• Reading your work leads to grasp three procedures (or phases, or experiments): primary questionnaire, secondary questionnaire, and a final language analysis; with each of the three having their own analysis and findings. Whether this is correct or not- the main body needs to be clearly recognized in terms of research vocabulary.

• When first mentioned in the text, consider clarifying the main sources of the (footbridge- trolley) scenarios – also known as the fat man scenario- and adding two lines to present what they are- even if they are publicly known, may be also referring to your Fig 2A - if possible.

• Table 1 seems to be a master table. It is referred to starting on page 12 but only appears on page 20, while constantly referring to its part between the two pages. To keep a more fluent readability, it may be more effective to present it earlier. An alternative solution is to break it into smaller tables following each related section. Perhaps, to recheck other figures and tables.

• Consider categorizing the (Discussion) section into more than one section, following the findings type, or any manner you find it sound and suitable for your work. It helps tracking the sequence of the whole work.

• The (Method) section - line 569 – normally comes at the first part of the text - before the main text body, to help the reader grasping the (big picture) of the research, and rationalize the overall structure of the experiment. It will be up to you to choose how and what to place in the method.

• Perhaps you would consider a transition between the introductions of the (steps – line 570) mentioned in this section – lines from 572-592, and their full explanation sections – starting at line 596. Perhaps also consider numbering these steps or use bullets to identify them.

Language adjustments:

o Perhaps you may consider shortening the length of the main title.

o It will help to review for typing errors (e.g. lines 90, 91 – to check the parentheses consistency).

o Consider the referencing style method (e.g. references 41 and 42 has a long list of authors, the style would shorten list).

o Consider not repeating titles and subtitles (e.g. Corpus generation – lines 124, and 572/596).

o May be to consider shortening the long subtitles (e.g. subtitles: “Detection is above chance and enhanced in morally engaging scenarios” – could be changed, for example to “Detection effects on moral scenarios”; or even “Source detection effects on moral scenarios”.

Reviewer #2: Overall this is an interesting paper that presents some very useful data on people’s approaches to moral decisions and their ideas about how AI might relate to these. I believe all the analyses etc. are correct, though I'm no expert on statistics. The discussion could be more theoretically rigorous in places, and how the results should best be interpreted seems to remain a little unclear. In general, it’s often not easy to tell what results were expected (and why) and which were more surprising (and why). There’s a lot of very interesting data, and one gets the feeling that there is even more of interest in it than has come out in this paper. The quantitative approach is effective, but it could have been even more interesting if there had been at least some qualitative component to reveal further insight into why people respond as they do and what they think it means.

Introduction:

The discussion of the literature shows that the area is complex and nuanced, with a variety of views and results. (On line 434 you refer to “the standard theory”: what is this, and is there one really?) It would be good if the paper took a clearer stance on certain key issues, since assumptions seem to be made about them throughout but are not always explicit. In particular, when and why is it crucial for people to be able to identify the source of something as AI? When does confusion about the source present a particular risk? Is mis- or disinformation generated by people any less dangerous, or is its generation by AI more a problem of quantity?

When might it be appropriate to prefer the judgement of an AI system? There seems to be an assumption that it never would be in moral contexts: if not, why not, and how do we identify moral contexts as such?

Given the topic of the paper, it would be acceptable to remain agnostic about some of the above questions, but in that case the discussion should be consistent about it.

The discussion of the literature could usefully indicate that much is uncertain or provisional. Assertions are made on the basis of one or very few studies. Ordinary replicability aside, everything involving AI is subject to rapid change, including perhaps people’s attitudes to it. This needs to be more explicitly acknowledged. LLMs’ justifications of their judgements are likely to be very much better since GPT5.x (especially where x >1) compared with GPT3.5. Any study in this area (including the present paper) can provide only a snapshot.

It might be useful to have a sub-heading at line 98, where the study itself starts to be described.

Overall structure:

It’s sometimes confusing that aspects of the experiment are mentioned before they have been described. Some of the description occurs in the Results section, while much more appears only in the Methods section at the end of the paper. It can make sense to put details about the methods at the end (which makes it very like an appendix), but then it’s important to make sure enough is included at the right times in the body of the paper to make it coherent. More comprehensive cross-referencing within the paper can also address this.

Points about specific lines in the text:

Line 85: “linguistic features play a significant role” — “Thus” before this seems misplaced because the previous text isn’t clearly about linguistic features. Really it needs the context of the discussion later in the paper.

99: Who were the participants? Social profile, age, gender etc.? These aspects could bear on their pattern of responses, e.g. relative to other studies. [I see this occurs in the method section: a cross-reference here would be useful.]

103: It’s confusing here (and sometimes elsewhere) to conflate deontological with emotional. Deontological responses would come from specific, often absolute moral principles; emotion seems on the whole more likely to relate to somewhat arbitrary and case-specific responses. Not everything that is non-consequentialist is deontological.

107: it would be useful to describe the experiment before mentioning it here and especially discussing the results. Similarly at line 114 “the footbridge problem” hasn’t been mentioned before.

Would it be rational for people to prefer judgements based on their source (whatever it might be) rather than on their perceived quality? Your position on this isn’t clear. If not, wouldn’t it be better if people never considered the source? In principle, shouldn’t people be considering the moral problem, making a considered judgement, and then assessing the judgement they are given by comparison with that? What do you mean by “strong contextual influences”? Is this desirable or not?

156-9: The “folk-psychology intuition” seems to be ungrounded for LLMs, prior to considering your results. One might expect that LLMs would mirror the dominant patterns in their training material, i.e. typical human judgements in a vast panoply of different situations; in which case there’s no reason why they would be more utilitarian, except to the extent that their operation is modified (if it is) by fine-tuning etc.

175-6: it would be good to know more about exactly how this incentivisation worked.

200-206: This point about agreement with the judgement vs agreement with the justification is important, but somehow often underplayed. It’s not always clear when “agreement” means with the one or the other, or perhaps both. What does it mean on line 205? How often do people agree with the decision but disagree with the justification, or vice versa, and are there interesting patterns in that?

214: do they agree more with the decisions as well as the justifications? Do the justifications tend to sway their agreement with a decision? (I’m not sure if your data can really show this.)

244-5: potential ambiguity of “significant” — the p-value is significant, but is the detection rate above 50% to a significant extent? (Cf. point about line 487 below.)

252-3: this point about “acceptability” seems to require a little more clarification as to whether the acceptability of the judgement is due to the linguistic quality (presumably of the justification) driving ideas about its source, or is determined irrespective of this. The phrasing here suggests the latter.

372-3: do you mean here the LLMs’ assumed or expected tendency, which according to the point around line 156-9 is incorrect?

407-8: again, it is wrong to conflate the personal and the deontological, if that’s what’s suggested here. Maybe you need a different term from “deontological” to capture this. Cf. point at line 103.

414: it seems the word “that” should be deleted in this line, unless I have misunderstood it.

417-21: perhaps here the tendency for the AI to give more logical explanation works in its favour — people are more likely to agree with these responses for complex cases because they are persuaded by the reasoning, regardless of the source? [I see this is similar to your point at 435-6.]

444-445: there could be an interaction here with the fact that the earlier model is less good at producing logical explanations in the complex cases?

449-50: but the findings you’ve just mentioned do seem to suggest a simple aversion to AI (or pro-human bias, which appears to be the same thing)? (Also, in this sentence, there are two groups of citation numbers: what do they each relate to? Why are there two different groups?)

487: it remained only a little above chance. How far above chance is useful/meaningful? Suppose it were only 51%? 54%? 58%? Is anything less than say 70% really meaningful?

508-9: Do you mean a cue to the judgement being utilitarian or it being AI-generated? These are not the same thing, even if people’s biases about them may tend to align.

514: in particular, might not generalise to more recent versions of GPT.

581-520: it’s not really clear here why the utilitarian/deontological dimension is treated as central, given that LLMs don’t necessarily engage in utilitarian reasoning, and that people may not be especially prone to recognise or reject it when they do.

535-541: why is there a tension or dissonance here? Or, what kind of tension is it? Suppose we agree that AI agents have no moral agency, this seems to be entirely compatible with their moral reasoning being good and legitimately persuasive (whether based on utilitarian or deontological principles). People’s moral reasoning is often terrible. People can often be immoral or amoral. It’s not clear that the judgements of an entity that has moral agency ought in general to be preferred, just because it has moral agency. What is the underlying theory here?

621: what are ambiguous answers and why were they removed? (Is ambiguity in moral judgement a bad thing? If so, why?)

**********

6. PLOS authors have the option to publish the peer review history of their article (what does this mean?). If published, this will include your full peer review and any attached files.

If you choose “no”, your identity will remain anonymous but your review may still be made public.

**Do you want your identity to be public for this peer review?**  For information about this choice, including consent withdrawal, please see our Privacy Policy.

Reviewer #1: No

Reviewer #2: No

**********

[NOTE: If reviewer comments were submitted as an attachment file, they will be attached to this email and accessible via the submission site. Please log into your account, locate the manuscript record, and check for the action link "View Attachments". If this link does not appear, there are no attachment files.]

To ensure your figures meet our technical requirements, please review our figure guidelines: https://journals.plos.org/plosone/s/figures

You may also use PLOS’s free figure tool, NAAS, to help you prepare publication quality figures: https://journals.plos.org/plosone/s/figures#loc-tools-for-figure-preparation.

NAAS will assess whether your figures meet our technical requirements by comparing each figure against our figure specifications.

# Author comment: Author response to Decision Letter 1

**DOI:** 10.1371/journal.pone.0353391.r002

3 Jun 2026

Dear Editor and Reviewers,

We would like to thank you for your time, careful review, and constructive feedback on our manuscript. We greatly appreciate your insightful comments, which have helped us clarify our methodology, reorganize our structure for better readability, and strengthen our overall discussion. Below, we provide a point-by-point response (in blue) to each of the suggestions provided by Reviewer #1 and Reviewer #2, detailing the specific revisions we have made to the manuscript.

Reviewer #1

1. "Consider identifying the research vocabulary clearly and in a sequential integrated manner, an (e.g. an Introduction - line 36 - cannot be followed by Results – line 123) even if you wish to keep your own titles."

"Reading your work leads to grasp three procedures (or phases, or experiments): primary questionnaire, secondary questionnaire, and a final language analysis; with each of the three having their own analysis and findings. Whether this is correct or not- the main body needs to be clearly recognized in terms of research vocabulary."

We added an “overview of the study” subsection to smoothen the transition from introduction to results.

“ Overview of the study

To systematically investigate human perceptions of AI moral decision-making, our study was conducted in three main phases. First, in the corpus generation phase, we collected judgments and textual justifications for a diverse set of moral and non-moral scenarios from both human participants and large language models (specifically, GPT-3.5 variants). Second, in the corpus evaluation phase, we presented these responses to new, independent groups of human evaluators who were tasked with identifying the authorship source (AI or human) and indicating their level of agreement with both the judgment and the justification. Finally, we performed extensive linguistic and semantic analyses, utilizing NLP techniques, predictive modeling, and SHAP interpretations, to isolate the specific textual features (such as formal reasoning cues and cost-benefit markers) that drove participants' detection accuracy and moral alignment. Full methodological details are provided in the Methods section.

“ 2. "When first mentioned in the text, consider clarifying the main sources of the (footbridge- trolley) scenarios – also known as the fat man scenario- and adding two lines to present what they are- even if they are publicly known, may be also referring to your Fig 2A - if possible."

We added this short description to the Introduction section (line 104-107):

"For instance, in the classic 'trolley problem,' participants decide whether to divert a runaway trolley to save five people at the cost of one (an impersonal dilemma). In the 'footbridge' variant, participants must decide whether to physically push a person off a bridge to stop the trolley (a personal dilemma; see Figure 2A for examples across scenario types)."

3. "Table 1 seems to be a master table. It is referred to starting on page 12 but only appears on page 20, while constantly referring to its part between the two pages. To keep a more fluent readability, it may be more effective to present it earlier. An alternative solution is to break it into smaller tables following each related section. Perhaps, to recheck other figures and tables."

We have broken the master Table 1 into smaller, section-specific tables (Table 1, Table 2, Table 3, etc.) and embedded them directly after the corresponding paragraphs. We thank the reviewer for this suggestion as it greatly improved the readability.

4. "Consider categorizing the (Discussion) section into more than one section, following the findings type, or any manner you find it sound and suitable for your work. It helps tracking the sequence of the whole work."

In the discussion, we added thematic subheadings to organize the flow of the findings: “Scenario complexity modulates preference for AI judgments, Belief-driven anti-AI bias overrides actual source, Preferred but unattributed: the LLM authorship paradox, In-group/out-group bias as an explanatory mechanism, Surface linguistic features drive detection alone, Semantic reasoning markers shape both detection and agreement, Limitations and moral-alignment caveats, Residual detectability despite humanization, Dissociating moral agency from moral judgment quality, LLMs as persuasive but unacknowledged moral advisors. “

5. "The (Method) section - line 569 – normally comes at the first part of the text - before the main text body, to help the reader grasping the (big picture) of the research, and rationalize the overall structure of the experiment... Perhaps you would consider a transition between the introductions of the (steps – line 570) mentioned in this section – lines from 572-592, and their full explanation sections – starting at line 596. Perhaps also consider numbering these steps or use bullets to identify them."

We follow a structure common in PLOS ONE publications, where detailed Methods appear after Results. To improve readability, we have added a Study Overview paragraph between Introduction and Results sections.

We have numbered the six steps in the Methods overview (Step 1: Corpus generation, Step 2: Corpus transformation, and so on) and added brief transition sentences between them to improve the flow and readability of this section. These changes can be found in the revised Methods section.

6. "Language adjustments: o Perhaps you may consider shortening the length of the main title. o It will help to review for typing errors (e.g. lines 90, 91 – to check the parentheses consistency). o Consider the referencing style method (e.g. references 41 and 42 has a long list of authors, the style would shorten list). o Consider not repeating titles and subtitles (e.g. Corpus generation – lines 124, and 572/596). o May be to consider shortening the long subtitles (e.g. subtitles: “Detection is above chance and enhanced in morally engaging scenarios” – could be changed, for example to “Detection effects on moral scenarios”;"

We thank the reviewer for their careful reading and constructive suggestions. We have addressed each point as follows.

We have revised the title. The new title now reads: "A Moral Turing Test: How Belief and Source Shape Detection of and Agreement with LLM Judgments." We believe this retains the key information while being more concise.

Regarding typos, we have proofread the entire manuscript. The parentheses inconsistency at lines 90–91 has been corrected, along with other minor typographical errors identified during this review.

References 41 and 42 have been reformatted to list the first six authors followed by "et al.," in accordance with PLOS ONE reference style guidelines.

We have renamed the relevant subsections to eliminate ambiguity. The Results subsection is now titled "LLMs replicate the moral framing effect " instead of corpus generation.

We have shortened the subtitles as suggested. For example, "Detection is above chance and enhanced in morally engaging scenarios" is now "Detection accuracy across scenario types"; "Agreement is shaped by both the scenario type and the belief about the source" is now "Agreement as a function of source and belief"; and so on. We believe these revised subtitles are more concise while preserving their informational content.

Reviewer #2

1. "Introduction: The discussion of the literature shows that the area is complex and nuanced, with a variety of views and results. (On line 434 you refer to "the standard theory": what is this, and is there one really?)"

We agree that "the standard theory" overclaimed consensus. We have replaced "the standard theory predicts that" with "dual-process accounts of moral cognition predict that" in the Discussion, attributing the framework explicitly to Greene and colleagues. The existing caveat to Kahane (ref. 53) in the following sentence already acknowledges that this framework is debated, and we have kept that caveat in place.

2. "It would be good if the paper took a clearer stance on certain key issues, since assumptions seem to be made about them throughout but are not always explicit. In particular, when and why is it crucial for people to be able to identify the source of something as AI? When does confusion about the source present a particular risk?... When might it be appropriate to prefer the judgement of an AI system?"

We have added a new paragraph in the Introduction laying out our working position:

“Taken together, these findings suggest the human perception of machine judgment can go in two opposite directions. A preference for AI may occur when machines are seen as authoritative sources of knowledge. In contrast, an aversion for AI might emerge from societal or psychological prejudices against machines, often stemming from the belief that machines lack agency and the capacity for compassionate or morally sound decisions (29). Ultimately, the importance of source identification depends heavily on the context of the interaction. Knowing whether content is AI- or human-generated becomes highly consequential when accountability is at stake, when the content is advisory rather than purely informational, and when users might calibrate their trust differently based on the author. While deferring to AI may be less problematic for pattern-recognition tasks with a clear ground truth, it becomes far more complex when the grounds for judgment are themselves contested. Moral preferences are precisely such a case: they capture ethically consequential scenarios (30, 31) where the basis for judgment is inherently subjective, making them a critical testing ground for examining the detection and acceptance of machine judgments by humans.”

3. "The discussion of the literature could usefully indicate that much is uncertain or provisional... LLMs' justifications of their judgements are likely to be very much better since GPT5.x (especially where x >1) compared with GPT3.5. Any study in this area (including the present paper) can provide only a snapshot." "514: in particular, might not generalise to more recent versions of GPT."

We have strengthened the Limitations paragraph to make the snapshot nature of the findings explicit. The paragraph now states that "the quality of LLM-generated moral justifications has increased substantially in more recent model families (e.g., GPT-4, GPT-5 and their successors). Our results should therefore be read as a snapshot tied to this generation of models. In particular, detection accuracy, the magnitude of the pro-AI bias in personal scenarios, and the persistence of the belief-driven anti-AI bias are all empirical questions that should be re-examined with newer model families." This flags both the generational limitation and the specific quantities that we expect to shift.

4. "It might be useful to have a sub-heading at line 98, where the study itself starts to be described."

We have added a "The present study" sub-heading immediately before the paragraph beginning "To investigate human perceptions [...]".

5. "Line 85: "linguistic features play a significant role" — "Thus" before this seems misplaced because the previous text isn't clearly about linguistic features. Really it needs the context of the discussion later in the paper."

We have deleted "Thus," and replaced it with "These findings suggest that ", which better reflects the actual logical relation between the preceding discussion of model-level alignment and the forward-pointing claim about linguistic features that we analyze later in the paper.

6. "99: Who were the participants? Social profile, age, gender etc.? These aspects could bear on their pattern of responses, e.g. relative to other studies. [I see this occurs in the method section: a cross-reference here would be useful.]"

We have added a cross-reference parenthetical immediately after the mention of 230 participants in the Introduction: "Full demographic details (age, gender, country) are reported in the Methods (see Participants)." This lets readers locate the demographic information without duplicating it in the Introduction.

7. "103: It's confusing here (and sometimes elsewhere) to conflate deontological with emotional. Deontological responses would come from specific, often absolute moral principles; emotion seems on the whole more likely to relate to somewhat arbitrary and case-specific responses. Not everything that is non-consequentialist is deontological." "407-8: again, it is wrong to conflate the personal and the deontological, if that's what's suggested here."

We have added a clarifying sentence right after the "deontological-like consideration" phrase in the Introduction: "We note that refusals in personal moral scenarios may reflect emotionally-driven aversion rather than principle-based (i.e., strictly deontological) reasoning; throughout the paper we reserve the term 'deontological' for principle-based judgments and use 'non-utilitarian' where the distinction matters." This explicitly separates the two constructs and signals the terminological convention we follow in the rest of the paper.

8. "156-9: The "folk-psychology intuition" seems to be ungrounded for LLMs, prior to considering your results. One might expect that LLMs would mirror the dominant patterns in their training material..."

We have rewritten the sentence to reframe the finding as an empirical test of the lay intuition rather than a direct challenge to a supposedly dominant theoretical claim. The sentence now reads: "Overall, our results empirically test, and do not support, the popular lay intuition that LLMs reason in a more utilitarian fashion than humans: in our data the LLMs we tested were no more utilitarian than human participants, and davinci-003 was in fact less so in personal moral scenarios." This positions the utilitarian-AI expectation as a folk belief to be tested, which is what it was.

9. "175-6: it would be good to know more about exactly how this incentivisation worked."

We have added a parenthetical right at the first mention of incentivization in the Results: "(a bonus of 5¢ per correct identification, yielding an average bonus of £1.46; see Participants in the Methods)". Readers now get the key quantitative detail inline without having to hunt for it in the Methods.

10. "200-206: This point about agreement with the judgement vs agreement with the justification is important, but somehow often underplayed. It's not always clear when "agreement" means with the one or the other, or perhaps both."

We have surfaced the operational definition of agreement early in the Results, rather than leaving it buried in the Methods. The inserted sentence reads: "Note that throughout the paper 'agreement' refers to the mean of two binary items collected for each response: agreement with the judgment (yes/no decision) and agreement with the justification (free-text reasoning), coded 1/0.5/0 for full agreement, partial agreement (agreement with one but not the other), and full disagreement (see Methods, Statistical evaluation). The two components move together in our data, but we flag divergences where they occur." This removes the ambiguity about which quantity "agreement" refers to.

11. "244-5: potential ambiguity of "significant" — the p-value is significant, but is the detection rate above 50% to a significant extent? (Cf. point about line 487 below.)"

We have rewritten the sentence to distinguish statistical significance from effect magnitude. The sentence now reads: "Overall, detection was statistically above chance but modest in absolute terms. In dv2 corpus (humans and davinci-002 justifications), participants correctly identified the source 64% of the time..." The word "significant" is no longer doing double duty, and the same clarification has been applied at the corresponding point later in the Discussion.

12. "252-3: this point about "acceptability" seems to require a little more clarification as to whether the acceptability of the judgement is due to the linguistic quality (presumably of the justification) driving ideas about its source, or is determined irrespective of this."

We have added a sentence making the causal structure explicit: "In our data, low-level linguistic features (length, typos, first-person pronouns) predicted participants' beliefs about the source but

Attachment Submitted filename: rebuttal_letter_response_to_reviewers_the_moral_turing_2026.docx

# Peer review: Decision Letter 1

**DOI:** 10.1371/journal.pone.0353391.r003

**Authors:** Stephen R. Milford, Murat Yildirim

24 Jun 2026

A Moral Turing Test: How Belief and Source Shape Detection of and Agreement with LLM judgments

PONE-D-26-10105R1

Dear Dr. Garcia,

We’re pleased to inform you that your manuscript has been judged scientifically suitable for publication and will be formally accepted for publication once it meets all outstanding technical requirements.

Within one week, you’ll receive an e-mail detailing the required amendments. When these have been addressed, you’ll receive a formal acceptance letter and your manuscript will be scheduled for publication.

An invoice will be generated when your article is formally accepted. Please note, if your institution has a publishing partnership with PLOS and your article meets the relevant criteria, all or part of your publication costs will be covered. Please make sure your user information is up-to-date by logging into Editorial Manager at Editorial Manager® and clicking the ‘Update My Information' link at the top of the page. For questions related to billing, please contact billing support.

If your institution or institutions have a press office, please notify them about your upcoming paper to help maximize its impact. If they’ll be preparing press materials, please inform our press team as soon as possible -- no later than 48 hours after receiving the formal acceptance. Your manuscript will remain under strict press embargo until 2 pm Eastern Time on the date of publication. For more information, please contact onepress@plos.org.

Kind regards,

T. Murat Yildirim, PhD

Academic Editor

PLOS One

Additional Editor Comments (optional):

When preparing the final draft, please address the minor issue raised by the reviewer (line 523).

Reviewers' comments:

Reviewer's Responses to Questions

**Comments to the Author**

1. If the authors have adequately addressed your comments raised in a previous round of review and you feel that this manuscript is now acceptable for publication, you may indicate that here to bypass the “Comments to the Author” section, enter your conflict of interest statement in the “Confidential to Editor” section, and submit your "Accept" recommendation.

Reviewer #2: All comments have been addressed

**********

2. Is the manuscript technically sound, and do the data support the conclusions?

The manuscript must describe a technically sound piece of scientific research with data that supports the conclusions. Experiments must have been conducted rigorously, with appropriate controls, replication, and sample sizes. The conclusions must be drawn appropriately based on the data presented.

Reviewer #2: Yes

**********

3. Has the statistical analysis been performed appropriately and rigorously?

Reviewer #2: Yes

**********

4. Have the authors made all data underlying the findings in their manuscript fully available?

The PLOS Data policy requires authors to make all data underlying the findings described in their manuscript fully available without restriction, with rare exception (please refer to the Data Availability Statement in the manuscript PDF file). The data should be provided as part of the manuscript or its supporting information, or deposited to a public repository. For example, in addition to summary statistics, the data points behind means, medians and variance measures should be available. If there are restrictions on publicly sharing data—e.g. participant privacy or use of data from a third party—those must be specified.

Reviewer #2: Yes

**********

5. Is the manuscript presented in an intelligible fashion and written in standard English?

PLOS ONE does not copyedit accepted manuscripts, so the language in submitted articles must be clear, correct, and unambiguous. Any typographical or grammatical errors should be corrected at revision, so please note any specific errors here.

Reviewer #2: Yes

**********

6. Review Comments to the Author

Please use the space provided to explain your answers to the questions above. You may also include additional comments for the author, including concerns about dual publication, research ethics, or publication ethics. (Please upload your review as an attachment if it exceeds 20,000 characters)

Reviewer #2: I'm now happy with the paper. Just one note: my comment about the original line 414, now line 523, still stands. As far as I can see, the word "that" is ungrammatical here and should be deleted from this line.

**********

7. PLOS authors have the option to publish the peer review history of their article (what does this mean?). If published, this will include your full peer review and any attached files.

If you choose “no”, your identity will remain anonymous but your review may still be made public.

**Do you want your identity to be public for this peer review?**  For information about this choice, including consent withdrawal, please see our Privacy Policy.

Reviewer #2: No

**********

# Editor report: Acceptance letter

**DOI:** 10.1371/journal.pone.0353391.r004

**Authors:** Stephen R. Milford, Murat Yildirim

PONE-D-26-10105R1

PLOS One

Dear Dr. Garcia,

I'm pleased to inform you that your manuscript has been deemed suitable for publication in PLOS One. Congratulations! Your manuscript is now being handed over to our production team.

At this stage, our production department will prepare your paper for publication. This includes ensuring the following:

* All references, tables, and figures are properly cited

* All relevant supporting information is included in the manuscript submission,

* There are no issues that prevent the paper from being properly typeset

You will receive further instructions from the production team, including instructions on how to review your proof when it is ready. Please keep in mind that we are working through a large volume of accepted articles, so please give us a few days to review your paper and let you know the next and final steps.

Lastly, if your institution or institutions have a press office, please let them know about your upcoming paper now to help maximize its impact. If they'll be preparing press materials, please inform our press team within the next 48 hours. Your manuscript will remain under strict press embargo until 2 pm Eastern Time on the date of publication. For more information, please contact onepress@plos.org.

You will receive an invoice from PLOS for your publication fee after your manuscript has reached the completed accept phase. If you receive an email requesting payment before acceptance or for any other service, this may be a phishing scheme. Learn how to identify phishing emails and protect your accounts at https://explore.plos.org/phishing.

If we can help with anything else, please email us at customercare@plos.org.

Thank you for submitting your work to PLOS ONE and supporting open access.

Kind regards,

PLOS ONE Editorial Office Staff

on behalf of

Dr. Murat Yildirim

Academic Editor

PLOS One
